# A colloidal quantum dot infrared photodetector and its use for intraband detection

**DOI:** 10.1038/s41467-019-10170-8

**Published:** 2019-05-09

**Authors:** Clément Livache, Bertille Martinez, Nicolas Goubet, Charlie Gréboval, Junling Qu, Audrey Chu, Sébastien Royer, Sandrine Ithurria, Mathieu G. Silly, Benoit Dubertret, Emmanuel Lhuillier

**Affiliations:** 1 Sorbonne Université, CNRS, Institut des NanoSciences de Paris, INSP, 75005 Paris, France; 2 Laboratoire de Physique et d’Etude des Matériaux, ESPCI-Paris, PSL Research University, Sorbonne Université, CNRS, 10 rue Vauquelin, 75005 Paris, France; 3 Synchrotron-SOLEIL, Saint-Aubin, BP48, 91192 Gif sur Yvette Cedex, France

**Keywords:** Electronic devices, Quantum dots

## Abstract

Wavefunction engineering using intraband transition is the most versatile strategy for the design of infrared devices. To date, this strategy is nevertheless limited to epitaxially grown semiconductors, which lead to prohibitive costs for many applications. Meanwhile, colloidal nanocrystals have gained a high level of maturity from a material perspective and now achieve a broad spectral tunability. Here, we demonstrate that the energy landscape of quantum well and quantum dot infrared photodetectors can be mimicked from a mixture of mercury selenide and mercury telluride nanocrystals. This metamaterial combines intraband absorption with enhanced transport properties (i.e. low dark current, fast time response and large thermal activation energy). We also integrate this material into a photodiode with the highest infrared detection performances reported for an intraband-based nanocrystal device. This work demonstrates that the concept of wavefunction engineering at the device scale can now be applied for the design of complex colloidal nanocrystal-based devices.

## Abstract (web summary)

The field of wavefunction engineering using intraband transition to design infrared devices has been limited to epitaxially grown semiconductors. Here the authors demonstrate that a device with similar energy landscape can be obtained from a mixture of colloidal quantum dots made of HgTe and HgSe.

## Introduction

Nanocrystals (NCs) are semiconductor nanoparticles with size-tunable optical features^1^. Interest for these materials has been driven at first by their bright luminescence, which has led to their integration as light source for displays^2,3^. Solar cells have also been quickly identified as a promising application for NCs^4^, thanks to the possibility to harvest the infrared (IR) part of the solar spectrum and to multi-exciton generation^5,6^. Solar cells were in this sense a first step toward NC-based IR photodetection^7^. More recently, material synthesis progresses, and more particularly the synthesis of mercury chalcogenides^8–10^, have allowed to significantly expand up to the THz, the range of wavelengths reachable with NCs^11^. Recent reports include continuous wavelength lasing with mercury telluride NCs^12^ as well as a significant breakthrough relative to the use of NCs for midwave-IR (MWIR) detection such as design of background limited detector^13^, demonstration of focal plane arrays, and intraband photoconduction^14,15^. Despite those insights, the driving technologies for IR detection remain based on epitaxially grown semiconductors.

Narrow bandgap semiconductors such as InSb and HgCdTe featuring interband transitions have been widely used as active materials for IR detection in the 3–5 and 8–12 µm atmospheric transparency windows. These materials nevertheless do not offer as much tunability as the doped heterostructure of III–V semiconductors, used in the so-called quantum well/dot IR photodetector^16,17^ (QWIP and QDIP, respectively) and type II superlattices^18,19^. Such heterostructures rely on intersubband transitions to provide absorption in the IR and have reached a high level of maturity. The introduction of quantum cascade detectors^20,21^ and unipolar barriers has allowed to develop the photovoltaic counterpart of QWIP and to reduce dark current. For colloidal materials, the maturity of the wavefunction engineering lags far behind. Among main achievements, one can cite the development of type I heterostructures to achieve high-brightness NCs^22^ and design of long-lived excitons in type II core–shell materials^23^. Even less work has been devoted to the concept of combining optical and transport properties in a material made of colloidal NCs^24,25^.

Designing complex functional heterostructures from NCs is nevertheless a critical point for the integration of NCs as active material for IR sensing, especially to compete with historical technologies^26^. New applications, in the short-wave IR and MWIR, such as LIDAR (light detection and ranging) detection, material sorting, assistance to night driving, and thermal management of buildings, require significant cost reduction, which are unlikely to occur from epitaxy-based mature technologies. Beyond the cost reduction, colloidal NCs also present significant advantages such as reduced Auger effect^27,28^ that should help in the quest of increasing the operating temperature of IR sensors.

Transport in NC arrays is usually done with a single NC population with a fine control of the size dispersity to avoid the largest particles to behave as trap states for the hopping carriers^29,30^. Recently, the concept of using a mixture of nanoparticles with on-demand electronic spectrum has appeared for the design of light-emitting diodes^24^ and solar cells^31,32^ or tune the average doping magnitude of NC arrays^33^.

Here we apply this concept to mid-IR photodetection and revisit the design of QWIP/QDIP. More specifically, we design a colloidal quantum dot IR photodetector (CQDIP), the colloidal counter part of QDIP. In practice, we demonstrate that such CQDIP can be obtained from a mixture of HgSe NCs, which present intraband absorption^14,15,34^ with non-degenerately doped HgTe NCs^35^. The careful choice of material sizes and proportions allows to optimize the photocharge extraction and to accelerate the photoresponse. We then integrate this metamaterial into the first NC-based intraband photodiode. The design of this diode takes, in particular, advantage of the recent development of unipolar barriers for colloidal NCs^35^. This allows us to report significant reduction in the dark current of the device compared to the material operated in a photoconductive configuration.

## Results

### Colloidal quantum dot IR photodetector

**Fig. 1:**
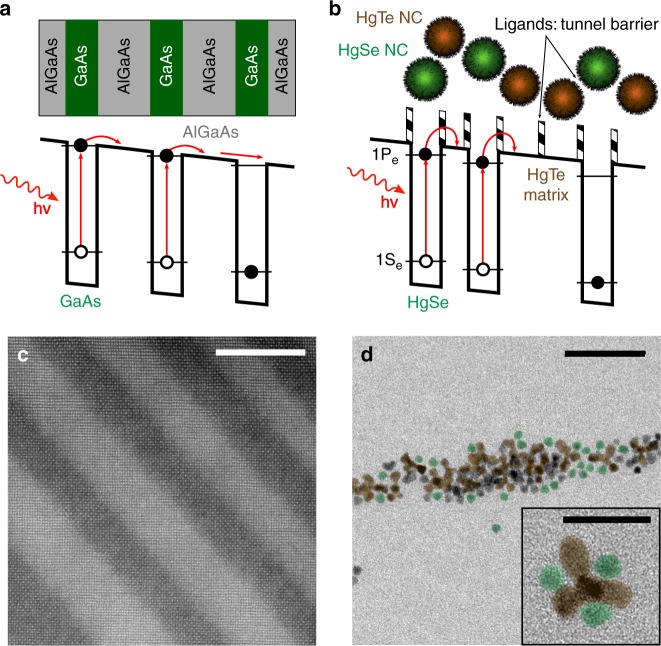
Principle of operation for a random colloidal quantum dot infrared photodetector (CQDIP). **a** Scheme of a standard GaAs/AlGaAs quantum well infrared photodetector (QWIP) band structure under polarization. **b** Scheme of polarized band structure of a random CQDIP consisting of HgSe nanocrystals (NCs) as absorber material and HgTe NCs as barrier material. **c** Transmission electron microscopic (TEM) image of an epitaxially grown GaAs/AlGaAs QWIP structure. GaAs quantum wells are shown in dark. Scale bar is 20 nm. **d** TEM image of mixed HgSe and HgTe NCs for random CQDIP fabrication. Some HgSe (resp. HgTe) NCs have been colored in green (resp. brown). Scale bar is 50 nm. Inset: higher-resolution TEM image showing a HgTe tetrapod along with three HgSe spheres. Scale bar is 20 nm

A traditional QWIP structure^16^ relies on series of GaAs quantum wells surrounded by barriers of AlGaAs, see Fig. 1a, c. The well width and barrier height are tuned to locate two confined states into the quantum wells: a ground state and an excited state, see Fig. 1a. The latter is generally degenerated with the top of the barrier to combine a strong oscillator strength and an efficient charge extraction. In a QWIP, the absorption relies on intersubband transitions in a degenerately doped GaAs quantum well. As a result, absorption selection rules prevent a normal incidence absorption and a diffraction grating needs to be etched at the surface of a pixel.

**Fig. 2:**
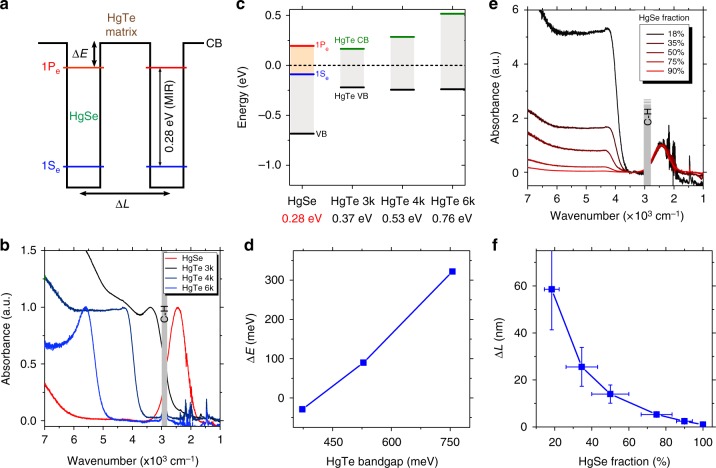
Design of the energy landscape of a colloidal quantum dot infrared photodetector (CQDIP). **a** Scheme of the band structure of a CQDIP showing two mid-infrared intraband HgSe nanocrystals (NCs) embedded in a HgTe NC matrix. Important CQDIP structure parameters are highlighted in bold: the energy difference between HgSe 1P_e_ levels and HgTe conduction band (CB), as well as the average distance between two HgSe wells. **b** Infrared absorbance spectra of the four materials used for this study: HgSe NCs; HgTe NCs with band-edge at 3000 cm^−1^ (HgTe 3k); HgTe NCs with band-edge at 4000 cm^−1^ (HgTe 4k), and HgTe NCs with band-edge at 6000 cm^−1^ (HgTe 6k). **c** Electronic spectra of the four materials used for this study, determined using a combination of X-ray photoelectron spectroscopic measurements and optical spectroscopy. Bandgap of each material is given in the *x* axis (intrabandgap is given for HgSe). Black dashed line is the Fermi level. **d** Energy difference $\Delta E = E_{{\mathrm{CB}}}({\mathrm{HgTe}}) - E_{1{\mathrm{P}}_{\mathrm{e}}}({\mathrm{HgSe}})$ between HgSe 1P_e_ level and HgTe CB as a function of the HgTe NC bandgap, extracted from **c**. **e** Infrared absorption spectra of HgSe/HgTe 4k NC solutions. Fractions relates to the proportion of HgSe particles in the mix. **f** Estimation of the average distance Δ*L* between two HgSe NCs for a given HgSe fraction, assuming a close-packing organization. Horizontal error bars are determined using the error of NC size from transmission electron microscopy and determining the impact of these fluctuations of the HgSe/HgTe ratio. Vertical error bars are determined using the HgSe fraction error

In the following, we propose a strategy to build a NC solid that presents a similar electronic landscape as a QWIP or QDIP, see Figs. 1b and 2a. Recently, it has been observed that doped II–VI NCs and especially mercury chalcogenides can present intraband absorption in the MWIR^15,36,37^. The observation of this transition in HgSe^38,39^ from the 1S_e_ to the 1P_e_ states of the conduction band is made possible by the self-doping^34^ of these particles resulting from the combination of their narrow bandgap nature and slight non-stoichiometry (i.e., cation excess). Here we use HgSe NCs presenting an intraband absorption with a peak at 2500 cm^−1^ (300 meV) at room temperature, see Fig. 2b. As the absorbing material is made from zero-dimensional spherical nanoparticles, no absorption selection rule is expected and this releases the need for a diffraction grating. In addition, dense films of NCs can be deposited and absorption in NC array is expected to be larger than for epitaxially grown semiconductor quantum dots. The absorption coefficient of HgTe NC thin film has actually been measured to be quite close to the one of the bulk^40^.

Transmission electron microscopy (TEM; Fig. 1d and Supplementary Fig. 1) reveals that the HgSe particles have a fairly spherical shape. Intraband photoconduction in an array of HgSe NC has already been demonstrated^14,15^, but the material suffers from three particular drawbacks. First, the photoresponse of the devices is fairly slow^15^ as soon as the doping level does not correspond to the fulfilling of the 1S_e_ state of the conduction band^14^. Then the current modulation under illumination is generally weaker^41^ than the one obtained with interband materials at the same wavelength. This is the counterpart of degenerate doping, giving access to intraband transition at the price of a larger dark current. Finally, films of HgSe NCs present a fairly low activation energy, meaning that cooling down the system does not allow to effectively reduce the dark current^42^.

For the barrier, we use HgTe NCs with a larger bandgap. HgTe has been chosen because it generally presents a larger mobility than HgSe^43^ and that its IR response is fast^44^. We thus expect that by combining the intraband absorption of the HgSe nanoparticles with the transport properties of HgTe we can combine the best of the two materials. In practice, we prepare a hybrid suspension of NCs by mixing two solutions of HgTe and HgSe with known concentrations. One can then calculate the HgSe NC ratio *x*_HgSe_ = *N*_HgSe NC_/*N*_NC_ using the volumes of individual NCs, see “Methods” and Supplementary Fig. 3. The nanoparticles are then dropcast from a hexane/octane solution and form a thin homogeneous film, which ligands are then exchanged to ensure a strong inter-particle coupling. We use 1,2-ethanedithiol (EDT) as ligand because of the strong affinity of thiol with mercury^45^. This ligand exchange step is crucial when building NC arrays since it allows to tune the inter-particular barrier length. This barrier ensures that the wave-functions stay mostly confined in the NC and has a typical height of 2 eV in the case of organic ligands. Switching from a 12-carbon aliphatic chain to a 2-carbon one (EDT) allows to reduce its thickness from ≈2 nm to ≈0.5 nm. This barrier reduction comes with a stronger inter-particle coupling and a rise of mobility from 10^–6^ to 10^–3^ cm^2^ V^−1^ s^−1^, typically^45^.

In a QWIP/QDIP, two main parameters have to be tuned to ensure charge extraction and charge transport: the energy of the excited state and the barrier width. In a mixture of HgSe and HgTe NCs, these two parameters are also tunable, see Fig. 2a. Here we choose to keep the same size of HgSe NCs along the study, so the difference of energy between the excited level of HgSe and the barrier (referred to as Δ*E*) can be tuned by changing the barrier height, hence by varying the size of the HgTe NCs. If too large bandgap HgTe NCs are used, the photocharge extraction will be poor since the excited state will not lead to drift transport in the continuum ($\Delta E \gg 0$). On the other hand, if a too low bandgap HgTe NCs are used, transfer from HgSe excited state to the barrier will be efficient, but thermal activation of carriers through the HgTe band edge will be large and a significant dark current contribution from the barrier will be observed. Here we have explored the impact of the tuning of the barrier height by using three sizes of HgTe NCs. Their respective excitonic feature appear at 6000 cm^−1^ (called HgTe 6k in the following), 4000 cm^−1^ (HgTe 4k), and 3000 cm^−1^ (HgTe 3k), respectively, see Fig. 2b.

It has been previously demonstrated that the electronic spectrum of such narrow bandgap NCs strongly depends on size and surface chemistry^34,43,46^, we thus choose to measure the exact spectrum of the involved NCs in absolute energy scale to determine their band alignment. We use a combination of photoemission and IR spectroscopy to locate the valence band and conduction band states of each material, see Fig. 2c and Supplementary Note 1 for methods. HgTe 3k and 4k have been selected because their conduction band is almost resonant with the 1P_e_ state (i.e., the excited state) of HgSe, see Fig. 2c, d.

Regarding the spacing of the wells, QWIP are generally designed in such a way that the residual tunnel coupling between the wells is weak. In the case of the HgSe/HgTe mixture, the NCs are randomly distributed and we can anticipate that enough HgTe NCs need to be introduced to prevent the percolative hopping between HgSe NC. The effect of the HgTe/HgSe ratio on the mixture spectrum is shown in Fig. 2e. We have estimated that by tuning the HgSe NC ratio from 100% to 18% we tune the effective barrier width (average distance between two HgSe NCs) from 1 to 60 nm, see Fig. 2f and “Methods”.

### Designing the CQDIP energy landscape

As HgTe NCs are introduced into the HgSe array, we observe three clear changes on the transport and phototransport properties. First, transport probed in a field effect transistor^47^ configuration (Supplementary Note 2) reveals that the effective material switches from degenerately n-type (i.e., no hole conduction and negative threshold voltage) to ambipolar (both hole and electron conduction), see Supplementary Fig. 5. The effective material obtained from HgSe/HgTe appears from a transport point of view as less doped than the pure HgSe, which is promising for the dark current reduction. This speculation is further supported by photoemission measurements, which show that the effective Fermi level moves toward the valence band as the HgTe is introduced, see Supplementary Fig. 2.

**Fig. 3:**
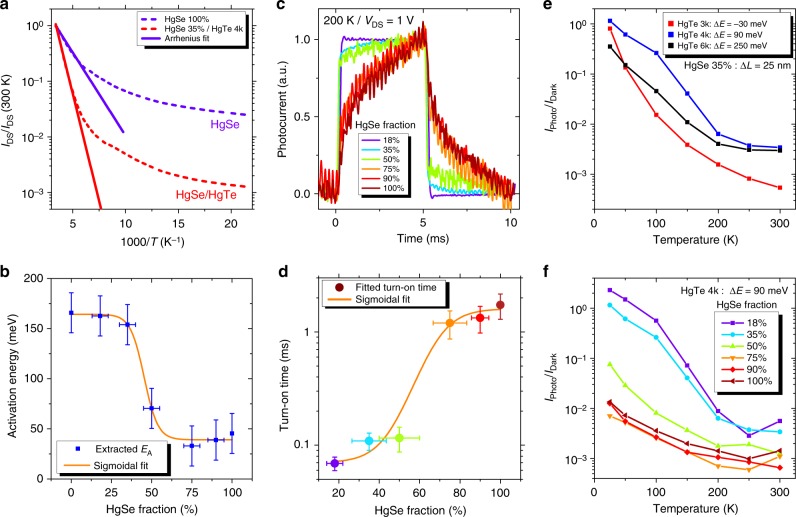
Transport and photoconductive properties of colloidal quantum dot infrared photodetector. **a** Evolution of dark current with temperature under *V*_DS_ = 1 V for two devices (pure HgSe and HgSe 35%/HgTe 4k). Current is normalized to its value at 300 K. The curves are fitted to Arrhenius law at high temperature. **b** Activation energy of HgTe 4k devices with different HgSe content, extracted from an Arrhenius fit of the cooling curves under *V*_DS_ = 1 V. Horizontal error bars are determined using the error on nanocrystal size from transmission electron microscopy and determining the impact of these fluctuations on the HgSe/HgTe ratio. Vertical error bars are set to 30 meV, which is an upper value of the fluctuation of the measured activation energy as we repeat the measure. **c** Photocurrent temporal traces for HgTe 4k devices with different HgSe contents, measured at 200 K under illumination by a 4.4-µm quantum cascade laser chopped at 100 Hz. **d** Turn-on times extracted from an exponential fit of the data presented in **c**. Color of each data point matches its curve from **c**. Vertical error bars represent s.d. **e** Effect of Δ*E*: evolution of the photocurrent over dark current ratio with temperature measured on devices made of HgTe 3k, HgTe 4k, and HgTe 6k with the same amount of HgSe (same Δ*L*). **f** Effect of Δ*L*: evolution of the photocurrent over dark current ratio with temperature, measured on six HgTe 4k devices (Δ*E* = 90 meV) with different HgSe contents (different Δ*L*)

In addition, the introduction of HgTe NCs leads to an increase of the thermal activation energy (Fig. 3a, b) and an acceleration of the photoresponse, see Fig. 3c, d. In this sense, results appear to be similar to what has been reported by Goubet et al. in the case of HgSe/HgTe core shell NCs^48^, but our strategy releases the constraint of an epitaxial growth of the shell of HgTe. Arrays of pure HgSe NCs present a weak thermal dependence, with the Arrhenius fit of the *I*(*T*) curve leading to activation energies around 30 meV. In other words, cryogenic operation for pure HgSe NC array barely reduces the dark current. Once HgTe is introduced, the activation energy is increased, see Fig. 3b, up to 160 meV. This value is similar to the one obtained for pure HgTe NC arrays; this suggests that in the mixture, the barrier driving the activation of the dark current is actually the thermal generation of charges within the HgTe NCs only. The latter value is very close to half the energy of the optical transition (300 meV), which is the expected value for an intrinsic semiconductor. From these observations, we can conclude that CQDIP behaves as a degenerately doped semiconductor (i.e., presence of intraband transition in the absorption) from an optical point of view but as quasi-intrinsic material from a transport point of view.

The time response of the detector is probed by resonantly exciting the intraband transition using a quantum cascade laser (QCL) operating at 2200 cm^−1^ (4.4 µm) and chopped at 100 Hz. When the HgSe content is high, we observe a time response of the order of 1 ms, see Fig. 3c, d. When the HgTe content is increased, the time response is shortened to values around 100 µs, limited by the configuration of the measurement set-up. To be sure that the observed photoresponse is indeed the result of intraband transition, we checked that an array of pure HgTe NCs does not present any photoresponse under MWIR illumination, see Supplementary Fig. 4. This acceleration of the device response (and in particular of the on-time) is the signature that the photoresponse switches from a bolometric effect (i.e., the increase of conductance is mostly due to heating of the NC film in the case of pure HgSe) to a photoconductive response (i.e., photon absorption and charge transport) in a quantum detector. Both the activation energy and time response clearly present an abrupt change of behavior when the HgSe content drops <50% and the two graphs are well fitted by a sigmoidal curve with a 45% and 60% threshold, respectively. This is consistent with the expected threshold for a three-dimensional percolation process.

In the following, we optimize the HgTe bandgap (i.e., the barrier height) and the HgTe content (i.e., the barrier width) leading to the optimal signal-to-noise ratio. For each sample, we choose to extract the photocurrent over dark current ratio (*I*_Photo_/*I*_Dark_) under the same illumination conditions. This value is a good figure of merit because it should scale as the detectivity. We found that the HgTe with a 4000 cm^−1^ band edge energy is the one presenting the highest photocurrent to dark current ratio, see Fig. 3e. While according to Fig. 2c, d, HgTe 3k would be a better choice in terms of barrier height, we only observe good performances at low temperature with this material. This reflects the fact that, if the HgTe bandgap is too small, thermal activation through the bandgap can occur, leading to an increase of dark current in the effective medium. At high temperature, the HgTe 6k is almost as good as the HgTe 4k because the poor band alignment is balanced by thermal activation. On the other hand, at low temperature, no activation from the 1P_e_ state to the conduction band of HgTe 6k is possible and less photocurrent is generated, leading to a reduction of the photo-to-dark ratio.

For a given barrier height (HgTe 4k is chosen in the following), we see that a large HgTe content favors the light-induced current modulation, see Fig. 3f. At very high HgTe content, the intraband absorption becomes weak and the modulation decreases, while no photocurrent is observed for the pure HgTe material, see Supplementary Fig. 4. The optimal ratio of intraband material is around 20–40%, which also corresponds to ratios for which the absorptions of HgTe and HgSe are of comparable magnitude, see Fig. 2e. This remains much higher than the absorption achieved in self-assembled quantum dot layer grown by epitaxy where the low density of quantum dot (≈10^11^ cm^−2^) leads to a very weak absorption.

### Intraband photodiode from CQDIP

In the next step, we integrate this CQDIP metamaterial as the absorbing layer of a photodiode. In a photodiode, the built-in electric field allows low bias operation, which is helpful to reduce the dark contribution of the current. While in the near IR many diode structures^49^ has been proposed, far less work has focused on the MWIR range^13,50^. A first obvious constraint comes from the need of transparent electrodes. In the near-IR, tin-doped indium oxide (ITO) and other transparent conductive oxides are widely used, but their absorption starts to be quite important in the MWIR range. As a result, thinner ITO layer has to be used, increasing the contact resistance. Here we choose another approach based on a thin (80 nm) metallic Al grid deposited on a sapphire substrate (Supplementary Note 4), with 70% transmission in the MWIR, see Supplementary Fig. 7. This strategy is more versatile and can be easily transferred to longer-wavelength ranges. Note that we have checked that no polarization selection rule results from the NC film itself, see Supplementary Note 5 and Supplementary Fig. 8.

**Fig. 4:**
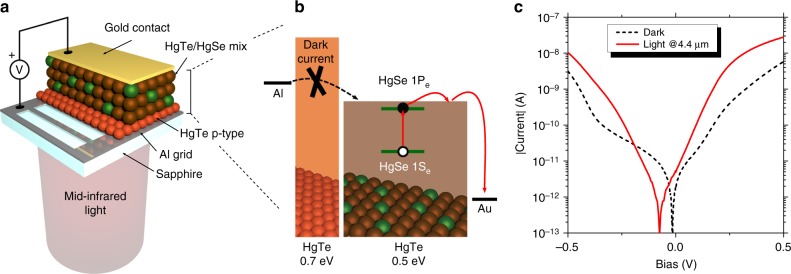
Design of an intraband photodiode from a colloidal quantum dot infrared photodetector. **a** Scheme of the device. Illumination is provided from the back side through a sapphire substrate and a patterned aluminum electrode allowing 70% of light transmission in the mid-infrared. **b** Band alignment diagram of the diode structure. HgTe 6k is used as a unipolar barrier to filter injection of dark current into the active HgSe/HgTe 4k layer. **c**. *I*(*V*) characteristics of the device measured at 80 K in the dark and in front of a 4.4-µm quantum cascade laser

A second challenge relates to the design of a carrier filtering layer required to obtain a rectifying behavior. n-type layer of ZnO and TiO_2_ is the most common strategy for solar cell design; however, the wide bandgap nature of these oxides ensures that a part of the photocurrent is also filtered^51^, which leads to a responsivity degradation. We rather use the strategy developed by Jagtap et al.^35^ who proposed to introduce a layer of small, wider bandgap HgTe NCs as a unipolar barrier. HgTe 6k appears to have its valence band almost resonant with HgTe 4k, while its conduction band is offset by >200 meV, see Fig. 2c, d. A thin layer of HgTe 6k can thus be used as an electron dark current filtering, see Fig. 4b. The complete diode is based on the following structure: Al_2_O_3_/Al/HgTe6k/HgSe-HgTe4k/Au, see Fig. 4a, b. Because this diode aims to be used as a unipolar diode, the hole dark current also needs to be suppressed and this is achieved by cooling the sample to cryogenic temperatures. We define the Al side as the 0 V reference in the following, and the *I*(*V*) curve of the system displays an asymmetric shape, see Fig. 4c. The choice of a thin layer of narrow bandgap material as HgTe 6k is also motivated by the fact that the Al side of the diode should provide the electrons needed to refill the quantum dots after extraction of their photo-exited electron. A too wide bandgap material would be detrimental from this point of view. In this sense, the magnitude of the barrier offset between the CQDIP and the unipolar filtering material sets the range of operating bias, as the energy drop over this barrier needs to stay small compared to the offset. In practice, the electric field should stay <70 kV cm^−1^ (1.8 V applied over the device, see Supplementary Note 6).

**Fig. 5:**
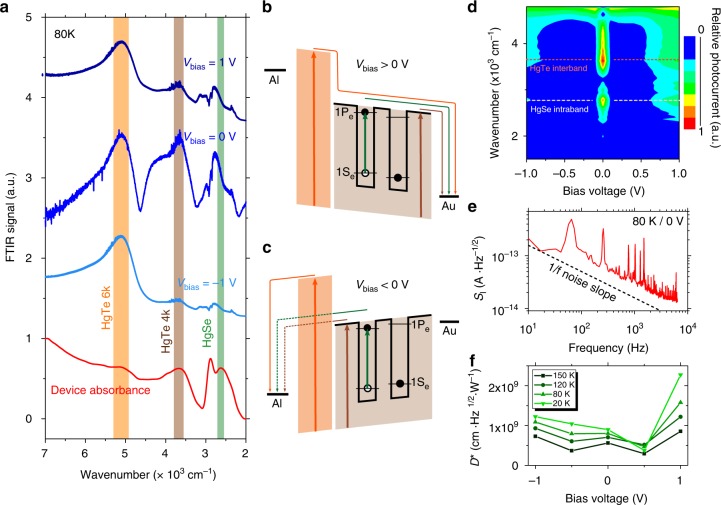
Spectral characterization of the intraband photodiode. **a** Photocurrent spectra and device absorbance measured at 80 K for several diode biases. Spectral contributions (maximum of absorbance) of HgSe (green), HgTe 4k (brown), and HgTe 6k (orange) have been highlighted. **b** Scheme of the band structure under positive bias. **c** Scheme of the band structure under negative bias. **d** Photocurrent map showing the relative contribution of the colloidal quantum dot infrared photodetector layer to the total photocurrent as the bias is changed. Measurement is made at 80 K with a 100-mV resolution, and each spectrum is normalized to the contribution of HgTe 6k. **e** Current spectral density measurement at 80 K and in short-circuit condition, showing a typical 1/*f* limited noise. **f** Specific detectivity of the device measured at 1 kHz

The investigation of the spectral response of the diode is very valuable to understand the diode behavior. Under broadband illumination (i.e., in a Fourier transform IR (FTIR)), photocurrent occurs from three different origins and can be written as *I*_photo_ = *I*_ph_(HgSe) + *I*_Ph_(HgTe4k) + *I*_ph_(HgTe6k). The photocurrent spectrum shows dramatic changes with bias, as shown in Fig. 5a. Under negative bias, only the unipolar barrier made of HgTe 6k is leading to a clear photocurrent signal: *I*_photo_ ≈ *I*_ph_(HgTe6k). This results from the filtering effect of the unipolar barrier that here prevents the extraction of photocharges generated in the HgSe/HgTe mixture, see Fig. 5c. Under positive bias, charge extraction is favored and we see an increase of the contribution from the HgSe/HgTe mixture. Under such bias, both the unipolar barrier and the HgSe/HgTe mixture contribute to the phototransport, see Fig. 5b. Figure 5d provides a map of the relative spectral photocurrent: *I*_Photo_/*I*_ph_(HgTe6k). This allows understanding under which conditions the diode actually performs as a good intraband detector. Again, we observe a clear asymmetry of the photoresponse with bias, which confirms the diode-rectifying behavior and positive bias operation as the magnitude of the intraband response is, as expected, larger under positive bias. The photovoltaic operation of the diode is also highlighted by the fact that 0 V operation leads to the largest relative contribution of the intraband to the photocurrent.

We notice that the intraband contribution of the photocurrent presents a small blue shift with respect to the material absorption, see Fig. 5a. This suggests that the photocharge extraction from the HgSe NCs is slightly non-optimal and that more energetic photocarriers are better extracted than the ones that are excited resonantly with the 1P_e_ state. This may have been expected from the photoemission measurement, see Fig. 2c, d, which predicts a 90 meV offset at room temperature. This also explains the relatively low responsivity measured under the QCL monochromatic excitation (see Fig. 4c and Supplementary Fig. 9d): the laser is matched to the red part of the intraband absorbance peak at room temperature, hence the spectral match of the photoresponse and the QCL is strongly affected by (i) the blue-shift of the absorbance with temperature and (ii) the observed blue-shift between the photoresponse and absorbance.

The responsivity of the diode measured under a blackbody with a 1.9-µm high-pass filter, where both HgTe 4k and HgSe contribute to photoconduction, reached a few mA W^−1^ under 1 V, see Supplementary Fig. 9 and Supplementary Note 7. The relative contribution of the interband and intraband to this response is discussed in the Supporting Notes, see Supplementary Figs. 9 and 10. The noise in the set-up has been found to be 1/*f* limited^52,53^, see Fig. 5e. Detectivity (i.e., signal-to-noise ratio) of 1.5 × 10^9^ Jones is achieved at 80 K and 1 kHz, see Fig. 5f. The fact that the device detectivity is maximum under 1 V polarization is again a confirmation that HgSe photocurrent contribution is larger under positive bias. Additionally, we have estimated the noise equivalent differential temperature of this device to be around 40 mK at 80 K and under 1 V bias, see Supplementary Fig. 10 and Supplementary Note 8. In terms of performance, this diode is presenting a detectivity that is factor two above the previous report obtained from HgSe NCs operated at the same wavelength and temperature^14^. This remains not as good as what has been obtained for pure interband transition NC-based devices^13^. This is mostly the result of lack of absorption due to the too thin layer of absorbing NCs. A comparison of the performance of the colloidal quantum dots with epitaxially grown quantum dots and quantum well IR sensors is also provided as Supplementary Table 1. Devices made with thicker films with enhanced light matter coupling^54–56^ will have to be developed in a second generation.

Another important feature of this diode is that the fast intraband photoresponse achieved with photoconductive devices is preserved, see Supplementary Fig. 11 and Supplementary Note 9. Under QCL excitation, pulse as short as 500 ns have been resolved. This is an important improvement of CQDIP compared to bolometer, the only low-cost alternative technology used for IR detection.

## Discussion

We demonstrate the design of a CQDIP metamaterial from a mixture of HgSe and HgTe NCs. This material combines intraband absorption in the MWIR with enhanced transport properties, that is to say lower dark current, higher activation energy, and fast time response. We also integrate this material into a photodiode and achieve a detectivity of 1.5 × 10^9^ Jones at 80 K with a sub-500 ns time response. Future development will have to increase the device responsivity by boosting absorption and propose new filtering barrier to allow for higher temperature operation.

## Methods

### Chemicals

Mercury chloride (HgCl_2_, Strem Chemicals, 99%), tellurium powder (Te, Sigma-Aldrich, 99.99%), selenium powder (Se, Sigma-Aldrich, 99,99%), trioctylphosphine (TOP, Cytek, 90%), oleylamine (Acros, 80–90%), dodecanethiol (Sigma-Aldrich, 98%), EDT (Fluka, 98.0%), lithium perchlorate (LiClO_4_, Sigma-Aldrich, 98%), polyethylene glycol (PEG, *M*_w_ = 6 kg mol^−1^), chloroform (VWR), ethanol absolute anhydrous (VWR), methanol (Carlo Erba, 99.8%), acetone (VWR), n-hexane (VWR), n-octane (SDS, 99%), and toluene (Carlo Erba, 99.3%) were used. All chemicals were used as received, except oleylamine, which was centrifuged before use. Mercury compounds are highly toxic and must be handled with special care.

### NC synthesis

*HgTe 3k*: 513 mg of HgCl_2_ is added to 60 mL of oleylamine in a 100 mL round flask. The solution is placed under vacuum and heated to 110 °C for 1 h. The temperature is then decreased to 100 °C and atmosphere is switched to argon. A total of 1.9 mL of TOP:Te (1 M) with 10 mL of oleylamine is added to the mercury solution. The solution color gradually turns to dark brown and the reaction is quenched after 3 min with the injection of a solution made of 1 mL of dodecanethiol and 9 mL of toluene. See Fig. 2b for optical spectrum and Supplementary Fig. 1 for TEM picture. The flask is cooled down and the NCs are then precipitated with ethanol. After centrifugation, the NCs are redispersed in chloroform. The washing step is repeated one more time before using the NCs.

*HgTe 4k*: 513 mg of HgCl_2_ is added to 60 mL of oleylamine in a 100 mL round flask. The solution is placed under vacuum and heated to 110 °C for 1 h. Then, the temperature is decreased to 80 °C and solution placed under Ar atmosphere. A total of 1.9 mL of TOP:Te (1 M) with 10 mL of oleylamine is added to the mercury solution. The solution color gradually turns to dark brown and the reaction is stopped after 3 min. A solution made of 1 mL of dodecanethiol and 9 mL of toluene is quickly added to quench the reaction. The NCs are then precipitated with ethanol. After centrifugation, the NCs are redispersed in chloroform. The washing step is repeated one more time. The solution is filtered with a 0.2-µm filter and redispersed in 6 mL of chloroform. See Fig. 2b for optical spectrum and Supplementary Fig. 1 for TEM picture.

*HgTe 6k*: 513 mg of HgCl_2_ is added to 60 mL of oleylamine in a 100 mL round flask. The solution is placed under vacuum and heated to 110 °C for 1 h. Then the temperature is decreased to 60 °C and the solution is placed under Ar atmosphere. A total of 1.9 mL of TOP:Te (1 M) with 10 mL of oleylamine is added to the mercury solution. The solution color gradually turns to dark brown and the reaction is stopped after 3 min. A solution made of 1 mL of dodecanethiol and 9 mL of toluene is quickly added to quench the reaction. The NCs are then precipitated with ethanol. After centrifugation, the NCs are redispersed in chloroform. The washing step is repeated one more time. The solution is redispersed in chloroform and filtered with a 0.2-µm filter. Two additional washing steps are applied with final redispersion in chloroform. See Fig. 2b for optical spectrum and Supplementary Fig. 1 for TEM picture.

*HgSe*: 500 mg of mercury acetate is dissolved in 10 mL of oleic acid and 25 mL of oleylamine. The solution is degassed under vacuum at 100 °C during 1 h. The atmosphere is switched to argon. At 110 °C, 1 mL of TOP:Se (1 M) is injected to the mercury solution. The solution rapidly turns from yellow to dark, indicating the formation of HgSe material. After 1 min, the reaction is quenched by addition of 1 mL of dodecanethiol and cooled to room temperature with a water bath. The NCs are then precipitated with ethanol. After centrifugation, the NCs are redispersed in chloroform. The washing step is repeated one more time. The solution is filtered with a 0.2-µm filter and redispersed in chloroform. See Fig. 2b for optical spectrum and Supplementary Fig. 1 for TEM picture.

### FTIR spectra

FTIR spectra are acquired using either a Brucker Vertex 70 or a Fischer Nicolet iS50 in ATR configuration. The spectra are averaged over 32 acquisitions and have a 4-cm^−1^ resolution.

### Estimation of HgSe fractions

In a first step, two solutions of HgSe and HgTe are diluted so that their absorbance at 415 nm is the same. They are mixed in a volumic ratio *x*_V_ = *V*_HgSe_/(*V*_HgSe_ + *V*_HgTe_). Assuming a 50/50 stoichiometry and similar atomic densities for the two nanoparticles, we can express the nanoparticle ratio as:

$$x_{{\mathrm{HgSe}}} = \frac{{V_{{\mathrm{HgSe}}}}}{{V_{{\mathrm{HgSe}}} + \frac{{v_{{\mathrm{QD}} - {\mathrm{HgSe}}}}}{{v_{{\mathrm{QD}} - {\mathrm{HgTe}}}}}V_{{\mathrm{HgTe}}}}}$$

where *v*_QD–HgSe_ and *v*_QD–HgTe_ are the nanoparticle volumes determined by TEM imaging. Assuming that one HgTe 4k nanoparticle is a big 14 nm tetrapod and HgSe a small 5 nm sphere, we use $\frac{{v_{{\mathrm{QD}} - {\mathrm{HgSe}}}}}{{v_{{\mathrm{QD}} - {\mathrm{HgTe}}}}} = 1/3$. Calculated HgSe ratios match reasonably well to energy-dispersive X-ray measurements, see Supplementary Fig. 3.

### Estimation of barrier length

The barrier length between two HgSe QD is the average distance between two HgSe NCs. In the scope of percolative transport, it can be thought as a one-dimensional situation. Hence, we define Δ*L* as

$${\mathrm{\Delta }}L = l_{{\mathrm{ligands}}} + d_{{\mathrm{HgTe}}} \times N_{{\mathrm{HgTe}}} = l_{{\mathrm{ligands}}} + d_{{\mathrm{HgTe}}} \times \left( {1 - \frac{{100}}{{x_{{\mathrm{HgSe}}}}}} \right)$$

where *l*_ligands_ is the distance between two packed HgSe NCs, *N*_HgTe_ is the average number of HgTe QDs between two HgSe QDs in one dimension, and *d*_HgTe_ is the diameter of one HgTe NC.

### Interdigitated electrodes for photoconductive devices

One-millimeter glass slides cut in half are cleaned by sonication in acetone and rinsed with isopropanol, then receive a 5-min oxygen plasma cleaning. An adhesion primer (TI-PRIME) is spin-coated onto each substrate and annealed for 2 min at 120 °C before AZ5214E resist is spin-coated and baked at 110 °C for 90 s. A MJB4 mask aligner is used to expose the substrates to ultraviolet light for 1.5 s through a lithography mask. Substrates are then baked at 125 °C for 2 min to invert the resist and flood-exposed for 40 s. AZ726 developer is used to develop the resist: the samples are dipped in the solution for 20 s before being rinsed in pure water for 10 s. Patterned substrates are dried and cleaned with 5 min of oxygen plasma to remove resist residues. In a thermal evaporator, 5 nm of chromium are deposited as an adhesion promoter before 80 nm of gold is evaporated. Lift-off is conducted in an acetone bath for at least 1 h. Electrodes are 2.5 mm long and spaced by 20 µm.

### Photoconductive devices

HgSe/HgTe solutions are redispersed in a 9:1 hexane:octane mix and drop-casted onto pre-patterned interdigitated electrodes. The film is allowed to dry for 10 s and capping ligands are exchanged toward EDT by dipping the device in a 1% EDT solution in ethanol for 90 s, then rinsing 40 s in ethanol. The process is repeated 5 times to fill the cracks created during the ligand exchange process and reach a 100-nm thickness.

### Activation energy

Devices are enclosed in a closed-cycle cryostat equipped with an occulted glass window. The samples are cooled down to 25 K, and current <1 V bias is measured with a Keithley 2634b source-meter. Temperature is measured with a Lakeshore 325 temperature controller using a calibrated sensor on the sample holder. Data are acquired through a homemade Labview software. Resulting *I*(*T*) curves are fitted to an Arrhenius model ($I_0 = Ae^{ - E_{\mathrm{A}}/kT}$) between 300 K and 100 K, allowing for the extraction of the activation energy *E*_A_.

### Optimization of photocurrent to dark ratios

Sample is enclosed in a closed-cycle cryostat. Illumination is provided through a glass window by a 4.4 µm QCL in quasi-continuous mode, optically chopped at 1 Hz. Bias and current are controlled with a Keithley 2634b source-meter through a Labview program. Starting at 300 K, an *I*(*V*) curve is acquired in the dark, then the current under 1 V bias is followed in time. The sample is kept in the dark for 10 s, then the laser is shone for 30 s, and finally the sample is in the dark for 90 s. This 1 Hz measurements allows for a very repeatable determination of *I*_photo_ and *I*_dark_. This sequence is repeated every 50 K steps. See Supplementary Fig. 4a for a scheme of the set-up.

We also check that pure HgTe devices do not show any photoresponse under QCL illumination, see Supplementary Fig. 4b.

Thickness dependence of responsivity and noise is discussed in Supplementary Note 3 and Supplementary Fig. 6.

### Time response at 100 Hz

In the same configuration as the *I*_photo_/*I*_dark_ measurement, the device is illuminated with a 100 Hz optically chopped QCL. One-V bias is applied by a Keithley 2634b source-meter, which also measures the average current. Current from the device is amplified by a Femto DLPCA 200 transimpedance amplifier, and fed into a Tektronix TDS 5034 oscilloscope. See Supplementary Fig. 4a for a scheme of the set-up.

### Diode fabrication

HgTe 6k (bandgap around 750 meV) is spin-coated on a patterned aluminum grid electrode. Ligand exchanged is conducted for 60 s in a 1% EDT solution in ethanol, then the device is rinsed in fresh ethanol. Three layers of HgTe 6k are deposited to achieve a 30–50-nm thickness. A HgSe/HgTe 4k mix solution with a HgSe ratio around 35% is then spin-coated on top of these layers, using the same ligand exchange process. Eight-to-12 layers are deposited to achieve a 200-nm thickness. Gold top contacts are evaporated through a shadow mask in a thermal evaporator. Supplementary Fig. 9a shows a picture of the device. Discussion about diode aging is provided in Supplementary Note 10 and Supplementary Fig. 12.

### Photocurrent spectra

The device is enclosed in a closed-cycle cryostat and cooled down to the desired temperature. The head of the cryostat is brought in the sample compartment of a Fischer iS50 FTIR spectrometer and illumination is provided from the backside of the device by the focalized Globar source through two ZnSe windows (one on the outer cryostat enclosure and one on the shield) and through a hole in the sample holder. The photocurrent is amplified using a Femto DLPCA−200 transimpedance amplifier, which also serves as a bias source. Output of the amplifier is sent back to the FTIR spectrometer through the ad hoc external detector adapter. All interferograms are normalized to the source spectrum, acquired with a flat-response DTGS detector in the same range of wavenumbers.

## Supplementary information

Supplementary Information

## Data Availability

The data that support the findings of this study are available upon reasonable request.
