# Supplementary material for: A colloidal quantum dot infrared photodetector and its use for intraband detection
Source: Nat Commun. 2019 May 9;10:2125. doi: 10.1038/s41467-019-10170-8 (PMC6509134; doi:10.1038/s41467-019-10170-8)
Supplement: Supplementary file 1 — Supplementary Information [file 41467_2019_10170_MOESM1_ESM.pdf]

Supplementary Information for

A Colloidal Quantum Dot Infrared Photodetector and its use for Intraband Detection

Clément Livache[1,2], Bertille Martinez[1,2], Nicolas Goubet[1,2], Charlie Gréboval[1], Junling Qu,[1] Audrey Chu[1], Sébastien Royer[1], Sandrine Ithurria[2], Mathieu G. Silly[3], Benoit Dubertret[2], Emmanuel Lhuillier[1*]

[1] Sorbonne Université, CNRS, Institut des NanoSciences de Paris, INSP, F-75005 Paris, France
[2] Laboratoire de Physique et d'Etude des Matériaux, ESPCI-Paris, PSL Research University, Sorbonne Université, CNRS, 10 rue Vauquelin 75005 Paris, France.
[3]Synchrotron-SOLEIL, Saint-Aubin, BP48, F91192 Gif sur Yvette Cedex, France

Table of content

1. Supplementary Figures 3
2. Supplementary Notes 12
3. Supplementary Table 17
4. Supplementary References 18

1. Supplementary Figures

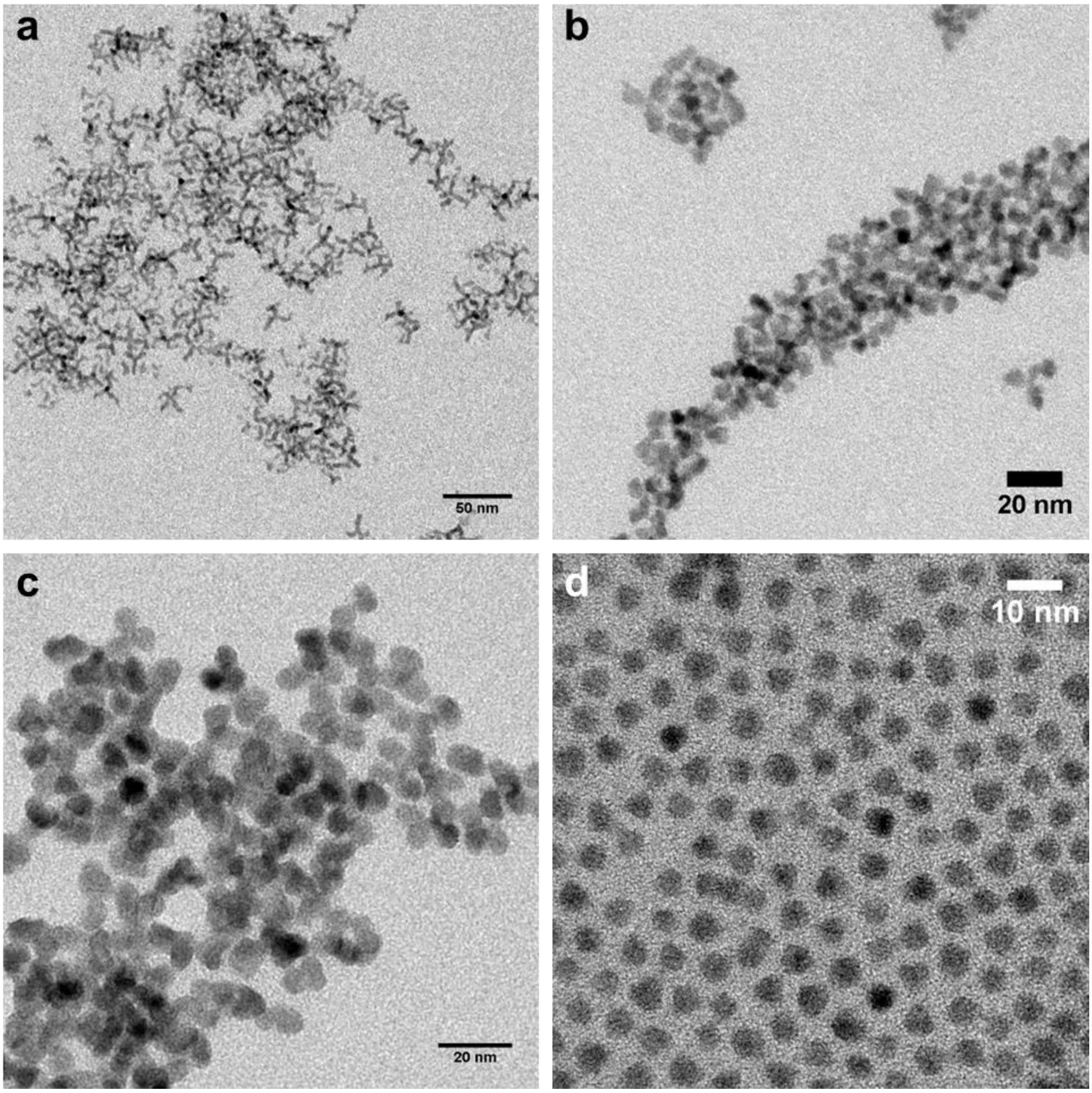

Supplementary Figure 1 Transmission Electron Microscopy (TEM) images of the nanomaterials used in this study. **a.** HgTe 6k nanocrystals, with bandgap around 6000 cm^{-1} (740 meV). Scale bar: 50 nm. **b.** HgTe 4k nanocrystals, with bandgap around 4000 cm^{-1} (500 meV). Scale bar: 20 nm **c.** HgTe 3k nanocrystals, with bandgap around 3000 cm^{-1} (370 meV). Scale bar: 20 nm. **d.** HgSe nanocrystals with intraband absorption around 2500 cm^{-1} (300 meV). Scale bar: 10 nm.

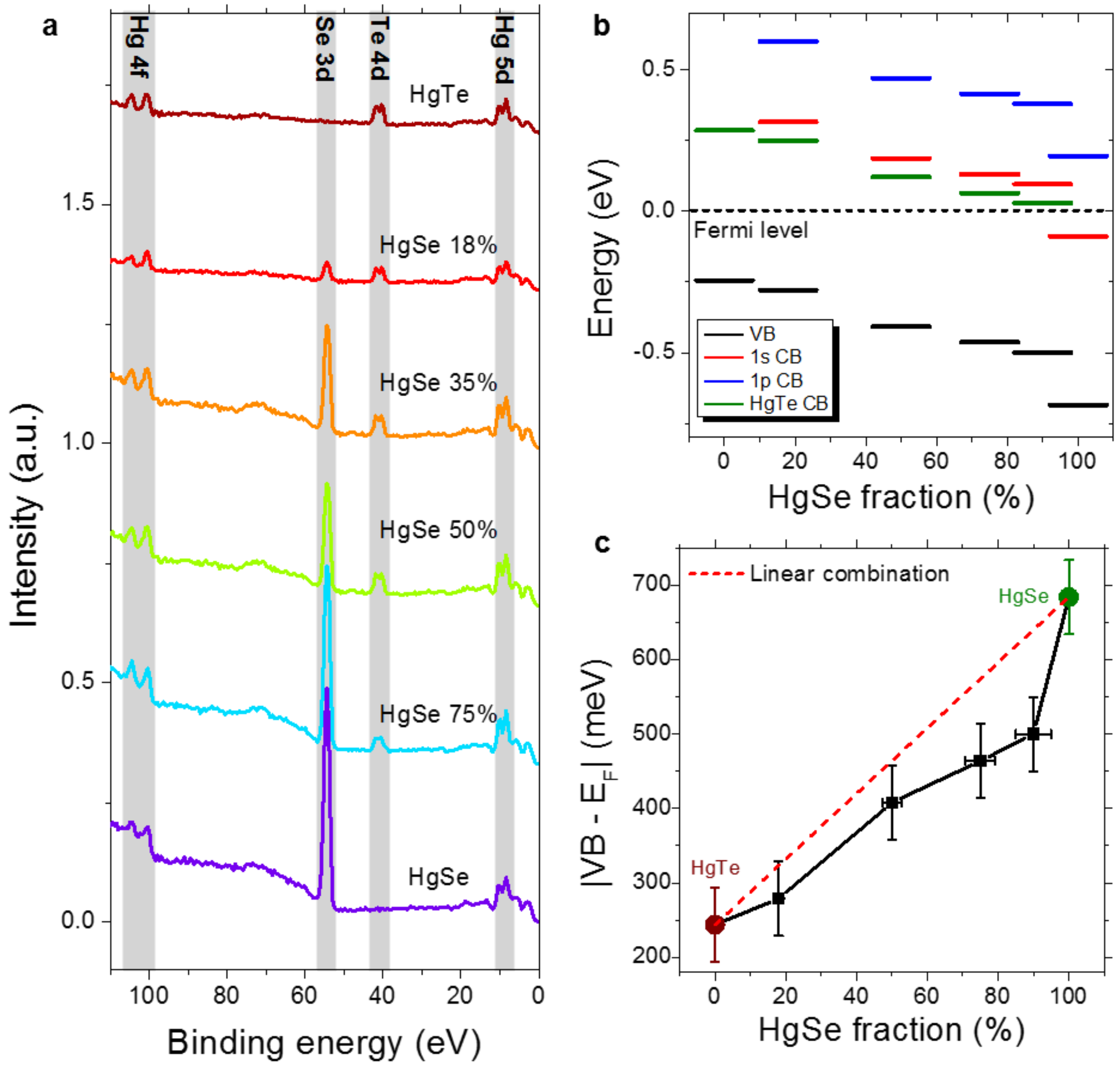

Supplementary Figure 2 X-ray Photoemission Spectroscopy (XPS) data on HgSe/HgTe 4k hybrid material. **a.** XPS overview showing Hg, Se and Te core levels peaks for different HgSe fractions. Data is acquired with 150 eV excitation. **b.** Reconstructed energy diagrams of HgTe 4k, HgSe and hybrid fractions. The Fermi level (E_F) is taken at 0 eV, valence band (VB) position is determined using XPS measurements and conduction band levels (CB, $1s_e$, $1p_e$) are placed using the bandgap value obtained from optical spectroscopy. **c.** Evolution of the energy distance between valence band (VB) and Fermi level (E_F) with HgSe fraction. Dashed red line is the linear combination $x_{HgSe}|VB - E_F|_{HgSe} + (1 - x_{Hse})|VB - E_F|_{HgTe}$. Horizontal error bars are obtained from size measurement error. Vertical error bars represent the confidence in the measured value.

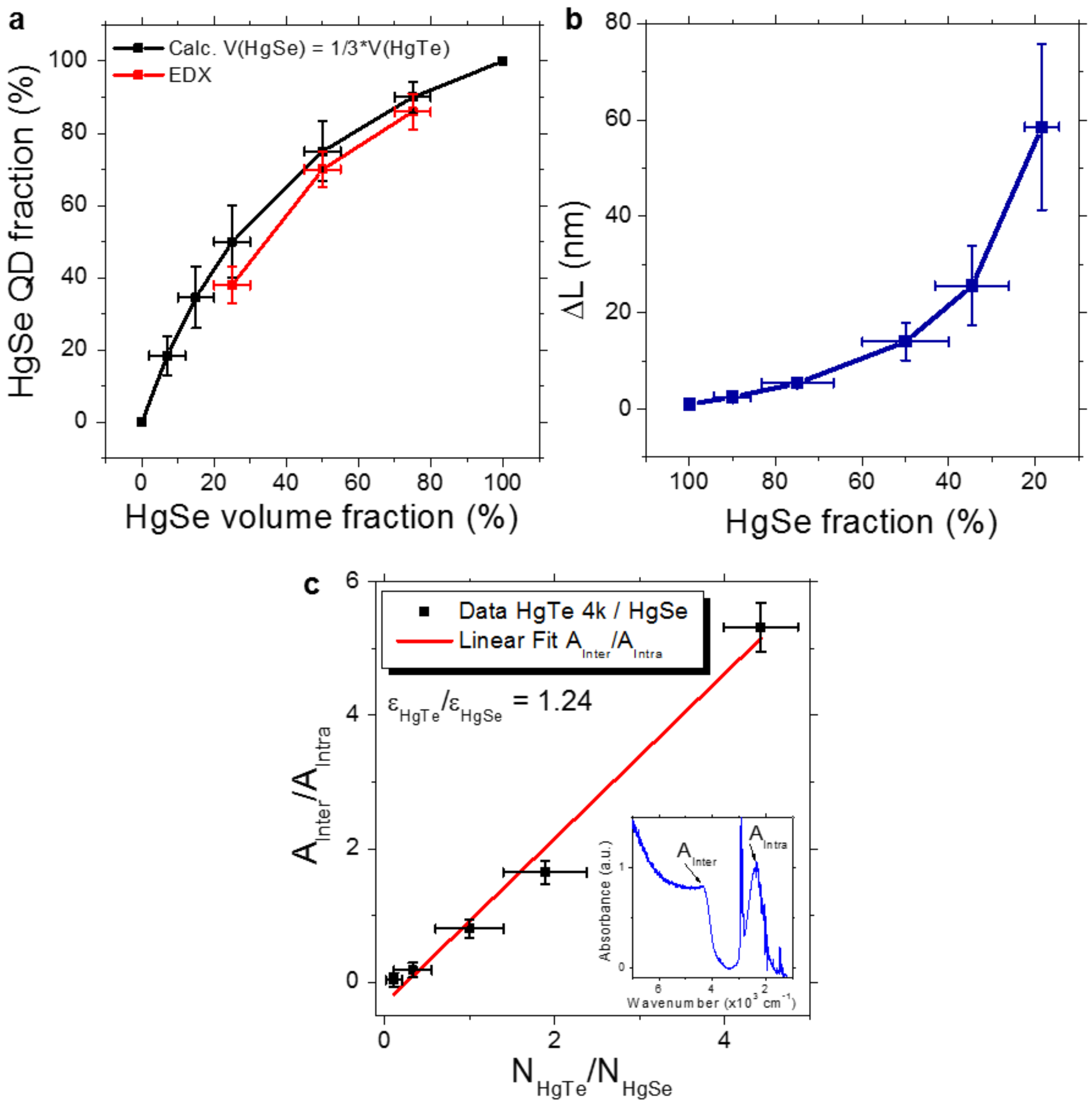

Supplementary Figure 3 Parameters derived from HgTe 4k / HgSe mixing. **a.** HgSe NC fraction derived from HgSe solution volumic fraction. See text for details about the calculation. In red: values measured on a thin film by Elemental Dispersive analysis (EDX) in a Scanning Electron Microscope (SEM). Horizontal error bars result from the error on initial solutions concentrations and vertical error bars from uncertainty on the NC volume ratio. **b.** Average length of the HgTe 4k barrier between two HgSe NCs. See text for details about the calculation. Vertical error bars result from errors (on d_{HgTe} and x_{HgSe}) propagation. **c.** Absorbance ratio between HgTe 4k (interband, taken around 4000 cm^{-1}) and HgSe intraband (taken around 2500 cm^{-1}) as a function of the NC ratio $\frac{N_{HgTe}}{N_{HgSe}} = \frac{x_{HgSe}}{1-x_{HgSe}}$, from data in Figure 2d. Horizontal error bars are calculated from error on the HgSe fraction. Vertical error bars are obtained from absorbance uncertainties. Inset: HgSe 50% spectrum with visualization of the two absorbance contributions.

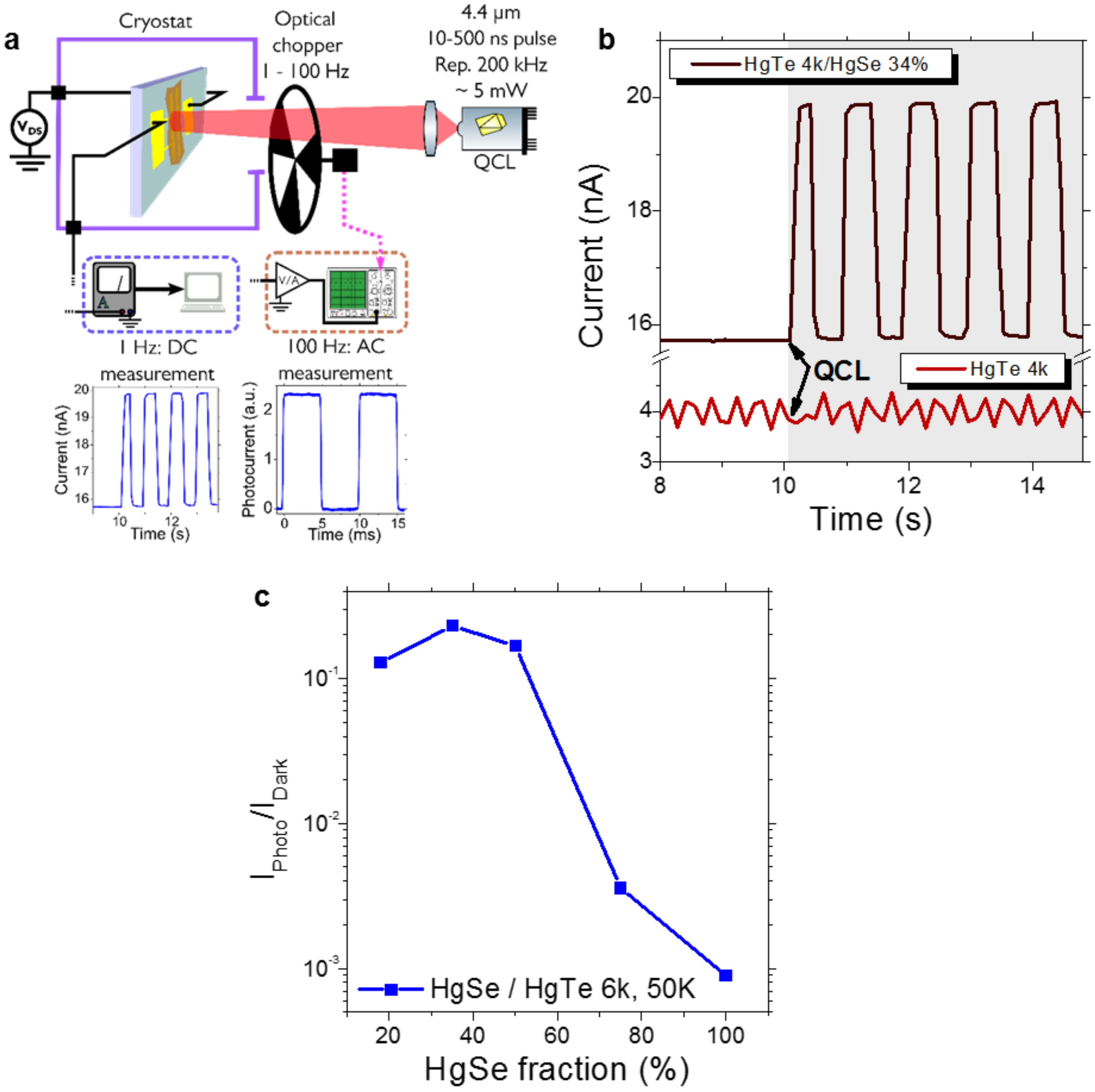

Supplementary Figure 4 Photoconductive device characterization **a.** Scheme of the experiments used to determine the I_{photo}/I_{dark} ratios and photoresponse dynamics for photoconductive devices. Sample are kept in a cryostat and illumination is provided by a 4.4 µm QCL. For the determination of I_{photo}/I_{dark}, the Quantum Cascade Laser (QCL) is chopped at 1 Hz and current is directly acquired through the source-meter (left graph). For the photoresponse dynamics, QCL is chopped at 100 Hz and the current is acquired with an oscilloscope through a transimpedance amplifier (right graph). **b.** Control experiment on pure HgTe 4k sample, showing no response to mid-infrared illumination compared to a 35% HgSe hydrid device. **c.** I_{photo}/I_{dark} ratios for several HgSe / HgTe 6k fractions at 50 K, showing a reduction of the modulation for low HgSe fractions due to low absorption (i.e. the loss of absorption is no longer balanced by the decrease of dark current).

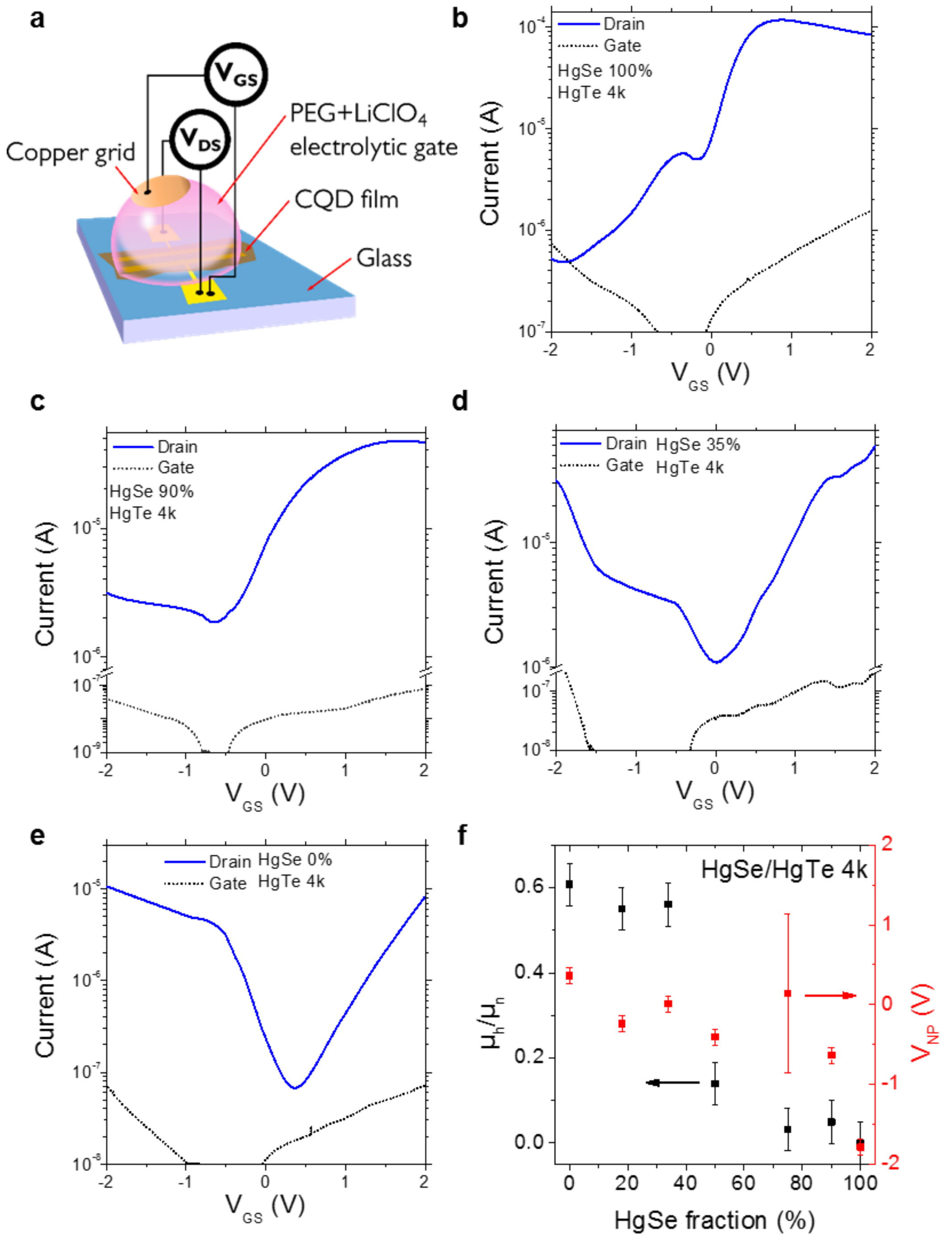

Supplementary Figure 5 Field Effect Transistor (FET) data for several HgSe ratios. **a.** Scheme of the electrolytic FET geometry. CQD: Colloidal Quantum Dots. **b-e.** FET transfer curves for pure HgSe **(b)**, 90% HgSe **(c)**, 35% HgSe **(d)** and pure HgTe 4k **(e)** films. All films are ligand-exchanged toward EDT and measurement is performed in air. Gate leak currents are given in black. **f.** Hole/electron mobility ratio (black) and neutral point (gate voltage corresponding to the minimum drain current, in red) for all the studied HgSe fractions. Error bars are determined by the hysteresis of the measurement and variations on several cycles.

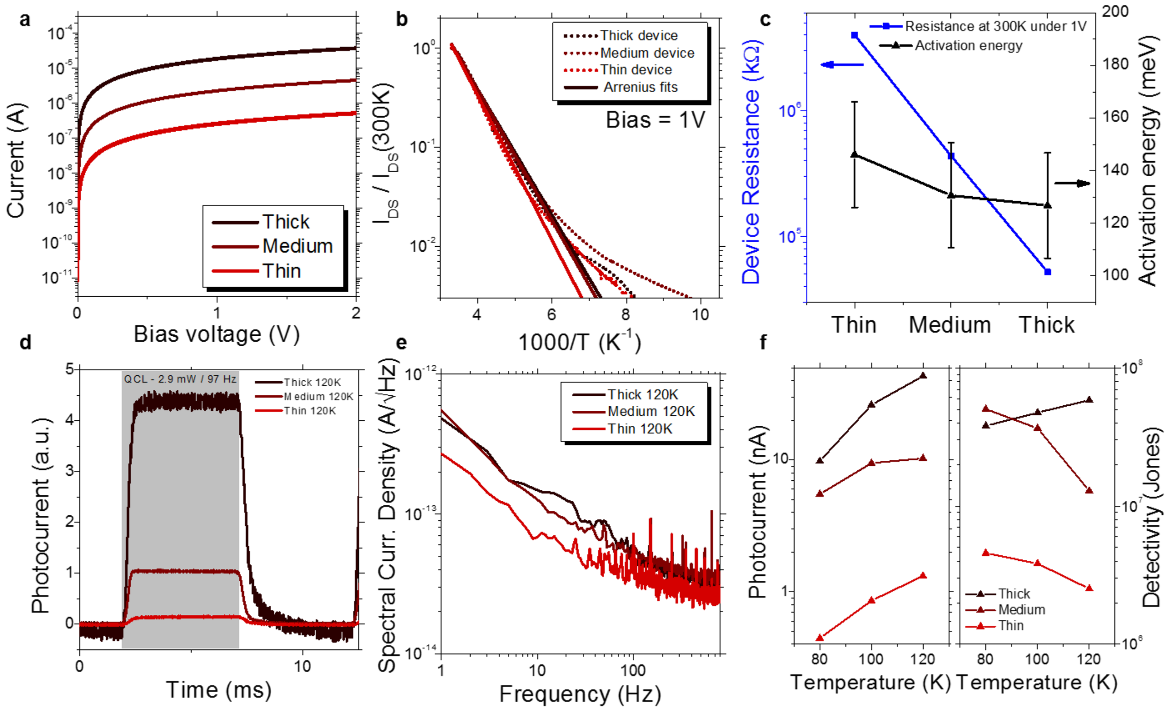

Supplementary Figure 6 Thickness dependence of the photoconductive device's performances. **a.** I-V characteristics of three devices with increasing thickness at room temperature. **b.** Normalized cooling curves of the three devices with associated Arrhenius fits. **c.** Extracted device resistances (under 1V bias, at 300K) and activation energies. Vertical error bars are set to 30 meV, the typical repeatability of the measurement. **d.** Photoresponse of the three devices to a 2.9 mW Quantum Cascade Laser (QCL) illumination chopped at 97 Hz. Devices are cooled at 120 K. **e.** Spectral current density of the three devices at 120 K, in the dark. **f.** Evolution of the photoresponse (under 2.9 mW QCL illumination) and detectivity with temperature, for the three devices.

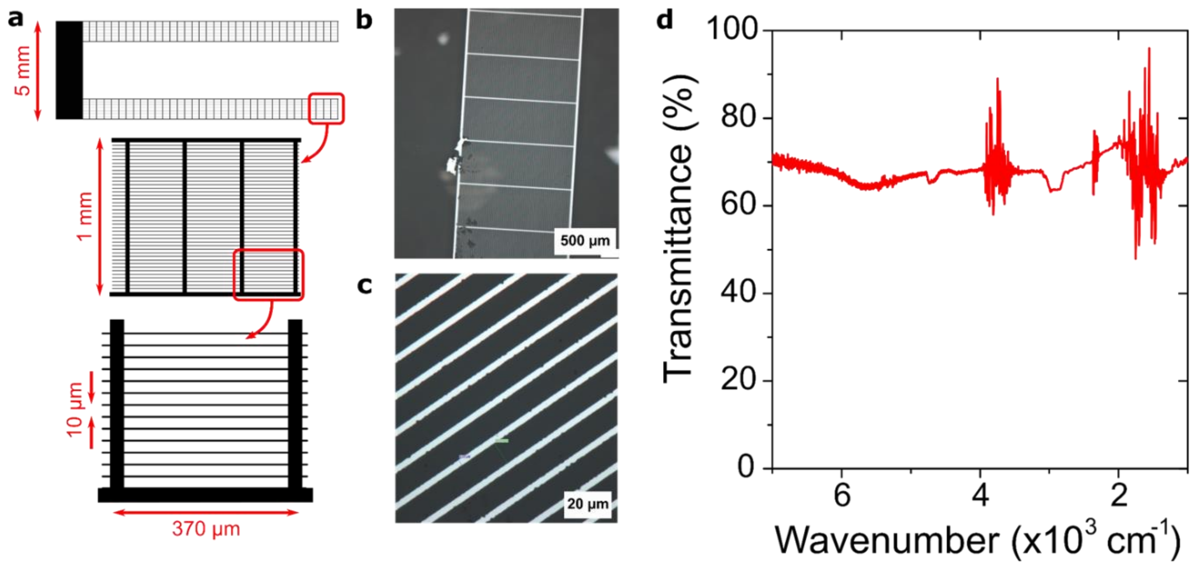

Supplementary Figure 7 Aluminum grid electrode used in the photovoltaic device. **a.** Sketch of the electrode at three levels of magnification, showing the grid structure. **b.** Optical microscopy image of one leg of a complete electrode on sapphire. **c.** Optical microscopy image of the 3 µm fine digits on a Al electrode on sapphire. **d.** Transmittance spectrum of the grid electrode in the near to mid infrared range, showing a relatively flat 70% transmission.

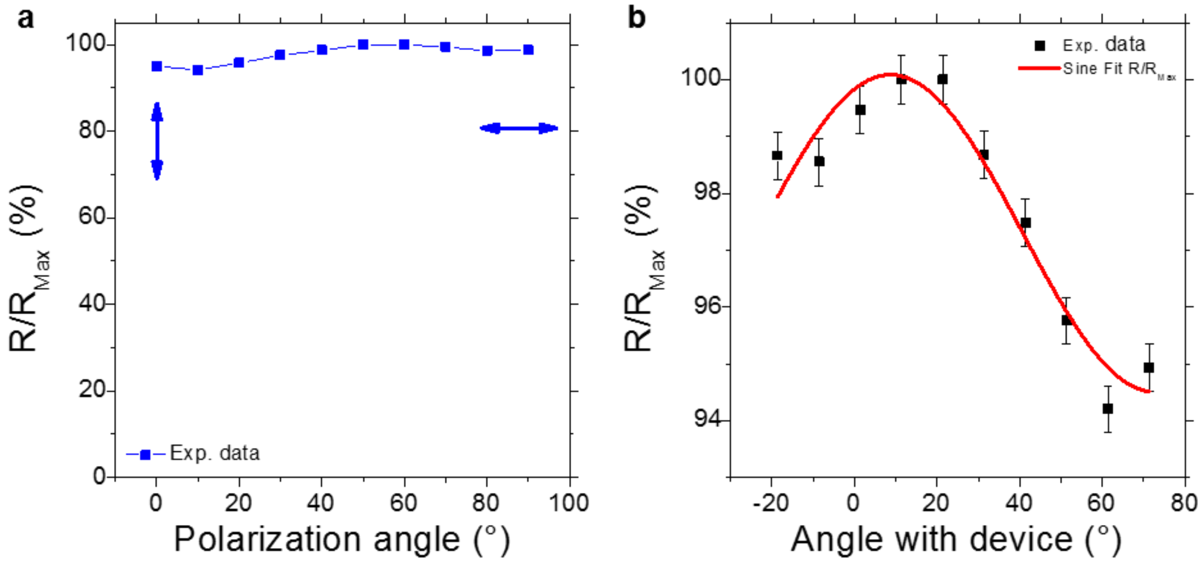

Supplementary Figure 8 Effect of light polarization on the device responsivity. **a.** Relative responsivity of the device when illuminated by a linearly polarized, 1.55 µm laser. **b.** Zoom on previous graph showing the small relative responsivity variations. Polarization angle is expressed relatively to the device orientation (0° : polarization along the device gold top-contact). Vertical error bars represent s.d.

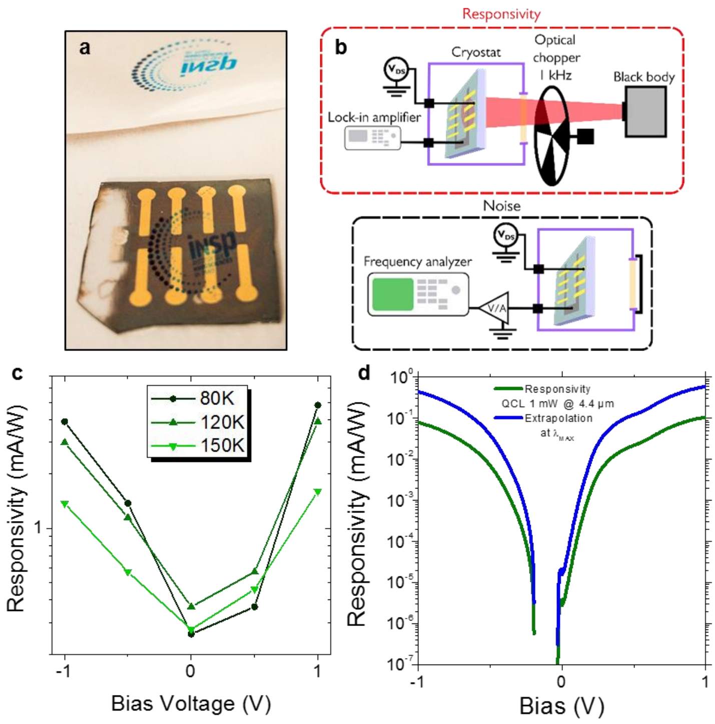

Supplementary Figure 9 Diode characterization. **a.** Picture of a device. A reflective device, as shown here, is characteristic of a good film quality. **b.** Scheme of responsivity and noise measurement. **c.** Responsivity of the device under 650°C black-body illumination. **d.** Responsivity at 80 K under a high-power Quantum-Cascade Laser (QCL) at 4.4 µm (green). In blue: extrapolated responsivity at λ_{MAX}=3.8 µm (using absorption ratio between 4.4 µm and 3.8 µm).

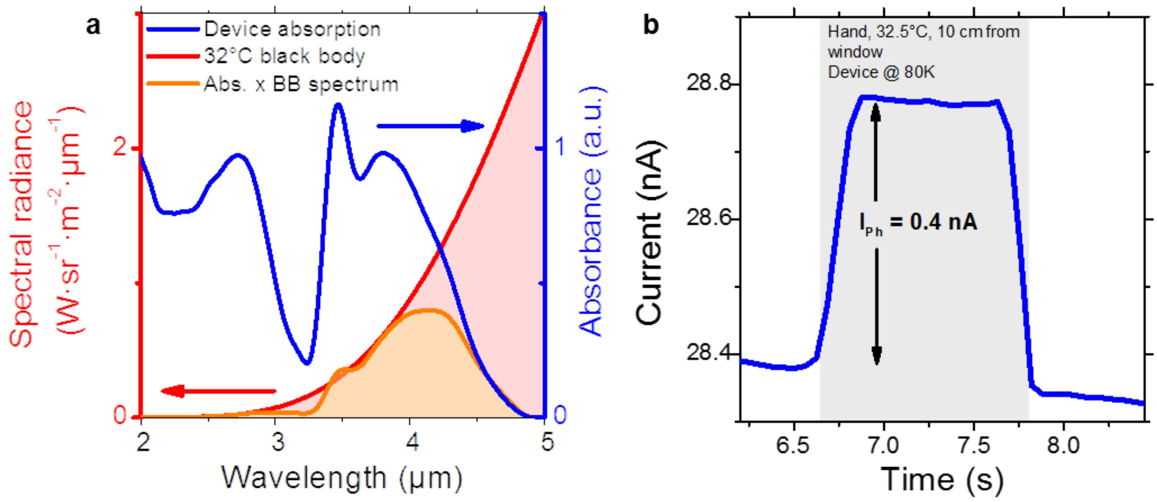

Supplementary Figure 10 Exposition of the device to a cold blackbody. **a.** Device absorption at 80 K normalized to the HgSe intraband feature (blue curve, right axis). Spectral radiance of a 32°C black body (red curve, left axis) and product of this spectrum with the device absorbance (orange curve). **b.** Electrical response of the diode to illumination by a hand (32°C black body) at 80 K under 1 V bias.

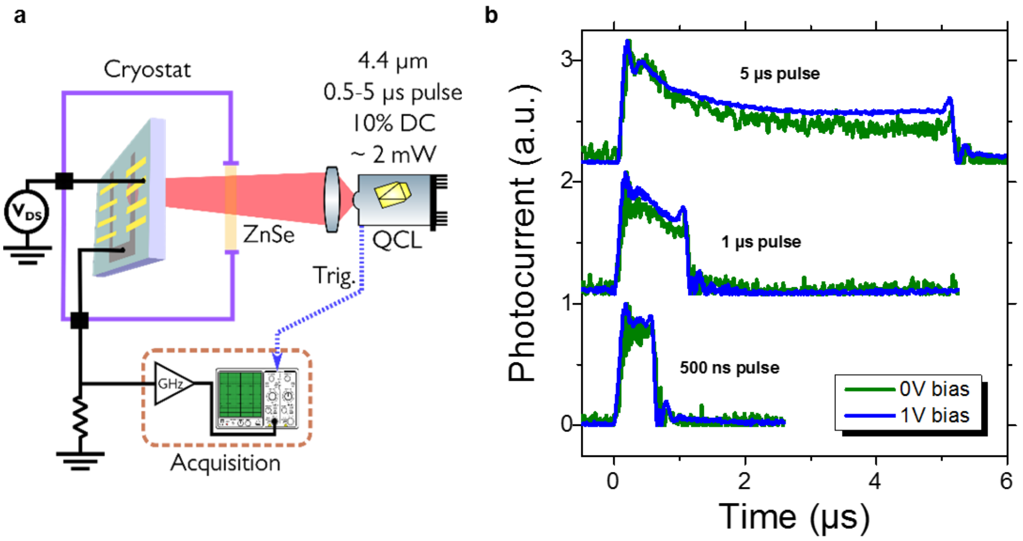

Supplementary Figure 11 Dynamics of MIR response of the diode device. **a.** Scheme of the MHz response measurement. QCL: Quantum Cascade Laser. **b.** Device response to short QCL pulses at 80 K, under zero and 1 V bias.

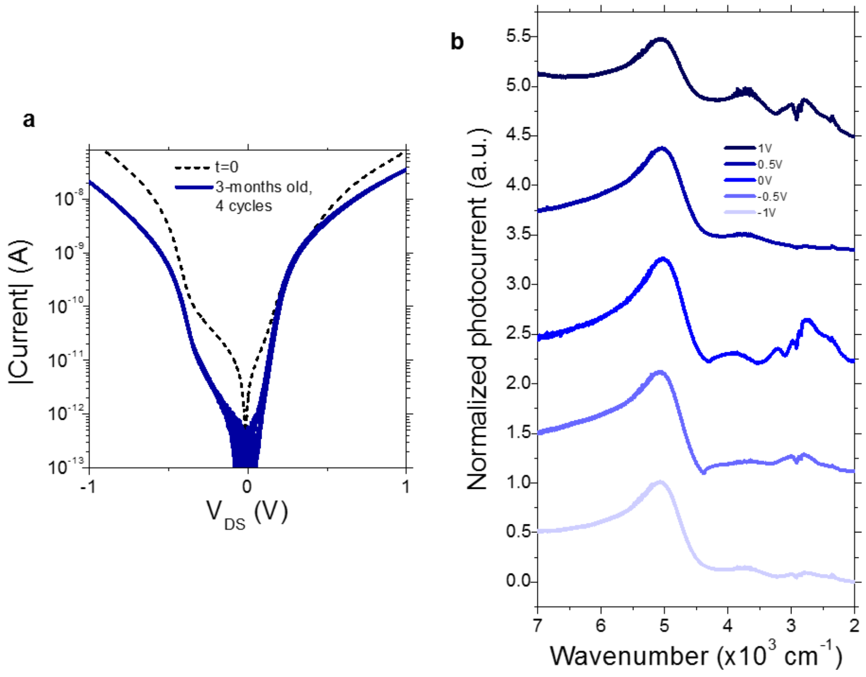

Supplementary Figure 12 Stability of the device after 3 months. **a.** I-V characteristic of the device in the dark, showing a slight reduction of the dark current after 3 months. 4 I-V cycles are displayed. **b.** Photocurrent spectra at 80 K on a the 3-month old diode.

2. Supplementary Notes

Supplementary Note 1: X-Ray Photoemission

XPS measurements have been conducted on the TEMPO beamline of Synchrotron SOLEIL, under ultra-high vacuum. The formula we used to determine binding energies (BE) is $h\nu_{\mathrm{exp}} = KE + BE$ where KE is the kinetic energy of the electron measured at the analyser, BE is its binding energy towards Fermi level and $h\nu_{\mathrm{exp}}$ is the photon energy.

Sample preparation. A silicon wafer is cleaned before 5 nm of chromium and 80 nm of gold are deposited by thermal evaporation. 8 mm x 8 mm pieces are cut and each substrate is drop-cast with HgTe/HgSe NCs in hexane/octane solution. Ligand exchange is performed using the same procedure as for transport devices, then the sample is degassed under ultra-high vacuum (10^{-10} mbar). The sample is introduced in the analysis chamber, where photoelectrons are detected at normal emission from the polarization vector **E**.

Photon energy. Photon energy is set to 150 eV, but $h\nu_{exp}$ has to be precisely measured. To do so, we look at the Hg 4f core levels of our material at the first and second order. Photon energy is then determined as $h\nu_{exp} = KE_{2nd} - KE_{1st}$ where KE_{2nd} is the kinetic energy of $Hg4f_{7/2}$ core level measured at second order and KE_{1st} is the kinetic energy of $Hg4f_{7/2}$ core level measured at first order.

Spectra calibration. We use a monocrystalline gold sample to calibrate the analyser, since its work function changes with the pass energy used. We measure the Fermi level of the gold sample as well as the photon energy for all the pass energies needed. We then set this Fermi level to zero as the sample is metallic and get the correction value to be applied to every spectrum.

Overview spectra. We take large scale spectra (overviews) in order to confirm the presence of mercury, selenium and tellurium in our samples. Spectra are acquired under 150 eV illumination with a step of 10 meV. All the observed peaks can be attributed to Hg, Se and Te core levels, see Supplementary Figure 2a. The measured kinetic energy is converted into binding energy using: $BE = h\nu_{exp} - KE$.

Valence band measurement. We determine the value of VB - E_F (energy difference from VB to Fermi level) by using 150 eV photon energy and looking at high kinetic energies electrons. We determine $h\nu_{exp}$ using the procedure described above. We measure KE_{VB} as the highest kinetic energy available and use the following formula to extract VB-E_F : $VB - E_F = BE_{VB} = h\nu_{exp} - KE_{VB}$. The measured value is a mean value of Fermi level to valence band energy distance in our effective medium, see Supplementary Figure 2b where we reconstruct the energy diagram of each material using XPS measurement in combination with optical spectroscopy.

Supplementary Note 2: Field Effect Transistor measurements

Electrolyte preparation: The electrolyte is prepared in a nitrogen-free glove-box. 0.5 g of $LiClO_4$ are mixed with 2.3 g of PEG (M_W = 6 kg.mol^{-1}). The resulting mix is heated at 170 °C for 2 h until the solution turns clear, then cooled down overnight before use.

FET Measurement: In a nitrogen-filled glove-box, a $LiClO_4$-PEG electrolyte is deposited on top of a photoconductive device, see Supplementary Figure 5a. The polymer gel is allowed to cool down overnight, then the transfer curve is measured under a 100 mV drain-source bias, with a 1 mV.s^{-1} gate bias sweep rate. Pure HgSe devices shows typical n-type transfer curves with a clear signature of Pauli blockade around zero gate bias, see Supplementary Figure 5b. Pure HgTe 4k devices show ambipolar behavior with an increase of conductivity on both p- and n- sides, see Supplementary Figure 5e. HgSe/HgTe 4k hybrid devices show intermediate transfer curves, with a monotonic transition from n-type to ambipolar, see Supplementary Figure 5c-d and Supplementary Figure 5f.

Supplementary Note 3: Thickness dependence of photoconductive devices

Samples have been prepared according to the procedure we report in the paper (deposition + solid state ligand exchange toward EDT), with 2 layers for the thin device (≈10-20 nm), 4 layers for the intermediate one (≈50-100 nm) and 7 layers for the thick one (≈150-200 nm). We use a 4.4 µm QCL to measure the devices responsivities.

In

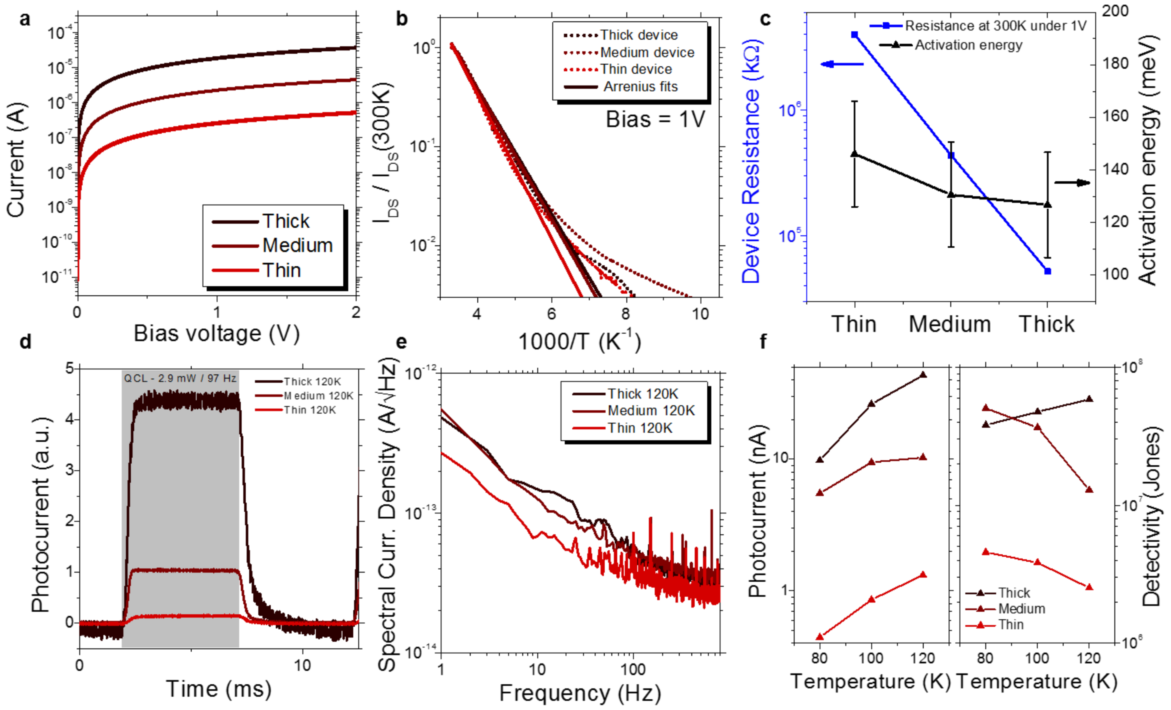

Supplementary Figure **6**a and b we provided the I-V characteristics of the three samples at room temperature as well as their cooling curve. Device resistance and activation energy are extracted form

previous data and are presented in

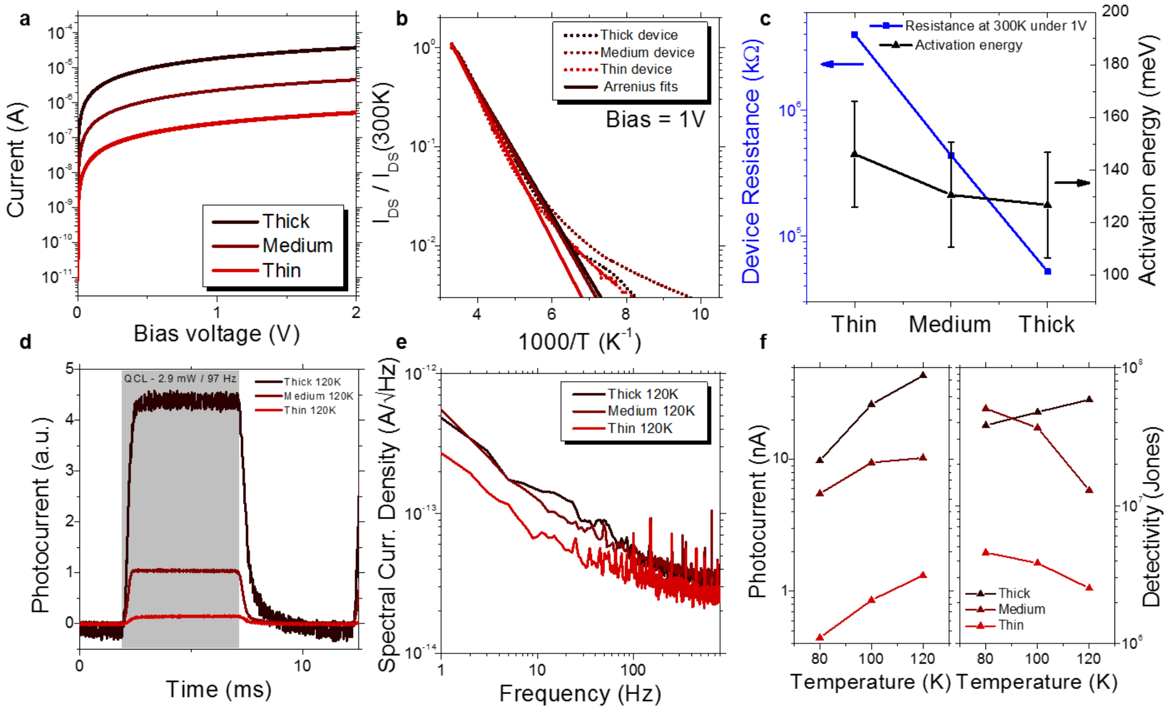

Supplementary Figure **6**c. The device resistance increases with thickness, which is expected for transport in disordered arrays of nanocrystals since thicker films come with a greater number of 3D percolation paths. Activation energy, on the other hand, is rather similar for the three devices as it only depends on the HgSe proportion in the initial solution. The responsivity of the three devices have then been measured between 80 K and 120 K using a 4.4 µm QCL chopped at 100 Hz, as well as the associated noise spectral density, see

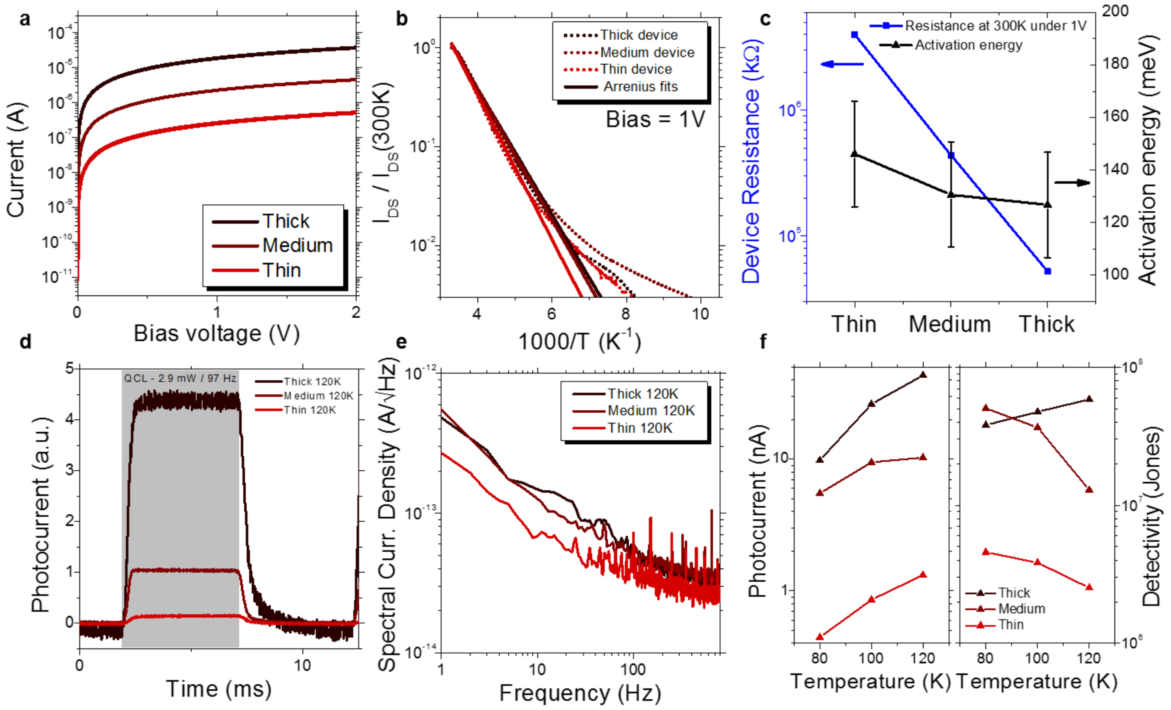

Supplementary Figure **6**d and e. There is a clear increase in responsivity with thickness (mostly due to higher absorption), when the noise level of the detector stays mostly unaffected as long as the NC film is of good quality (*i.e.* no cracks, no rough spot).

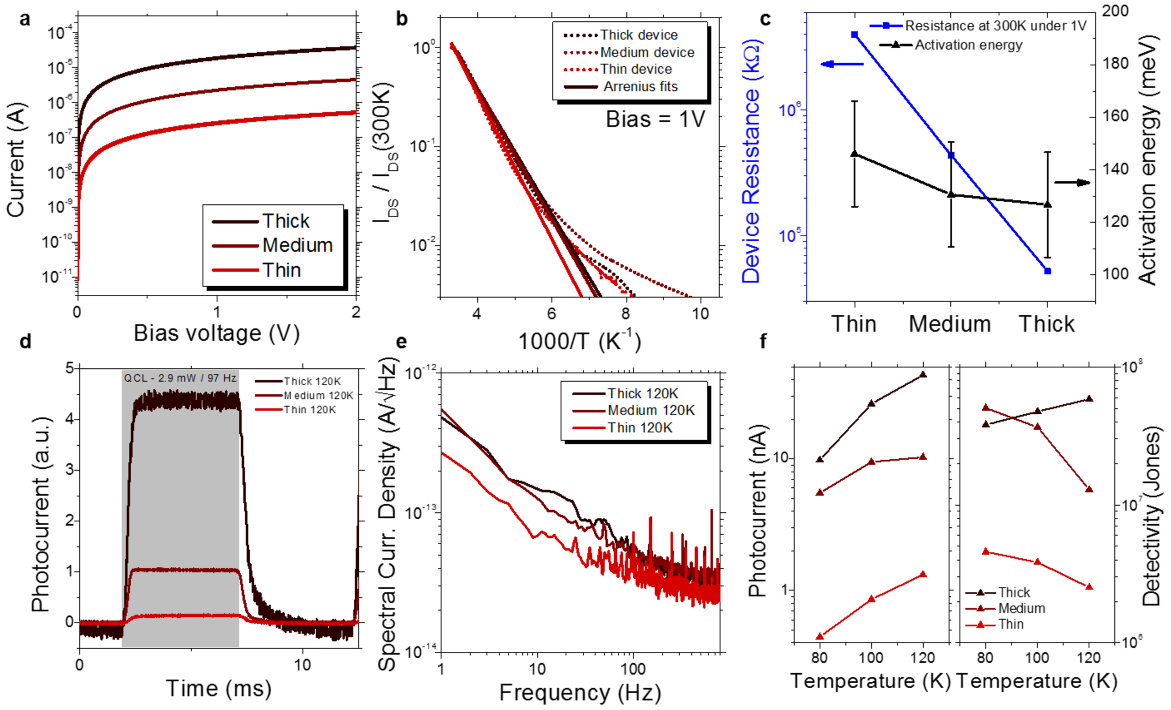

Supplementary Figure **6**f presents the evolution of responsivity and detectivity with temperature.

However, when it seems clear that film thickness should be maximized to get the highest detectivity in our devices, one should remember that when embedding such a film into a vertical device (such as a diode), the film quality is of utmost importance. Indeed, in order to prevent shortcuts and/or excessive noise, the NC film have to be extremely smooth, homogeneous over a large area and pinhole-free. Hence, there is a trade-off between thickness and film quality.

Supplementary Note 4: Al grid electrode

Sapphire substrates (2x2 cm²) are sonicated in acetone and cleaned with acetone and isopropanol before being exposed to an oxygen plasma for 5 min. Adhesion promoter (TI Prime) is spin coated on the substrate and annealed at 120 °C for 2 min. Then, the UV sensitive photoresist is spin coated on the substrate and is baked at 110 °C for 90 s, then a MJB4 mask aligner is used to expose the slides to UV light through a quartz mask (1.5 s exposition). After a 125 °C bake for 2 min and a 40 s flood exposure, substrates are developed using AZ726 developer for 20 s and rinsed with distilled water. After a 5 min plasma cleaning, 80 nm of aluminum is deposited using a thermal evaporator. Lift-off is conducted overnight in an acetone bath. The electrode is a U-shaped, 1 mm thick line patterned

with a grid to allow light transmission, see Supplementary Figure 7. The overall transmission of this electrode is around 70% in the target range of wavelengths.

Supplementary Note 5: Light polarization effect

We investigated the effect of polarization on the diode's responsivity, see Supplementary Figure 8. A horizontally-polarized 1.55 µm laser diode is used to illuminate the sample kept in a cryostat at 80 K. A $\lambda/2$ waveplate is used to rotate the light polarization and the photocurrent is measured using a Keithley K2634b source-meter. Illumination power has been measured for each polarization to ensure that any observed effect is not due to a change of incident power. Power variations stay below 3%.

While there is a slight effect of polarization orientation on the device responsivity the relative variation of around 5% is well below what is expected for traditional epitaxial QWIP devices, where the selection rules strongly depend on the incident light polarization. Since the illumination is provided from the back of the diode (through the Al mesh electrode), we believe that those slight variations might be due to diffraction effects through the fine digits of the Al electrode, see Supplementary Figure 7.

Supplementary Note 6: Limitations of the HgTe 6k barrier and device stability under bias

With a 200 meV offset between HgTe 4k and HgTe 6k and assuming a 30 nm-thick barrier, the critical electrical field for the barrier to become triangular is $F_C = 70$ kV/cm. Assuming a 250 nm total thickness (HgTe 6k layer + HgSe/HgTe 4k layer), this critical field is reached under 1.75 V bias. To prevent any damage to the diodes and to ensure the correct operation of the HgTe 6k unipolar barrier, those didoes need to be operated in the -1 to 1 V ranges of biases. Bias voltages above 2 V will eventually lead to electrical shorts.

Supplementary Note 7: Detectivity measurements

The device is enclosed in a closed-cycle cryostat and cooled down to the desired temperature. Supplementary Figure 9 provides the experiments schematics. Illumination is provided from the backside of the device through two ZnSe windows (one on the outer cryostat enclosure and one on the shield) and through a hole in the sample holder. The source is an Omega BB-4A calibrated blackbody operating at 650 °C and placed 30 cm away from the sample. The flux coming from the blackbody is filtered with a Ge window (1.9 µm cut-off high pass filter) and chopped at 1 kHz. Photocurrent from the sample is measured using a Zurich Instruments MFLI lock-in amplifier which also serves as a bias source.

Noise measurement: device is biased using a 1.5 V battery and a potentiometer. Current from the device is amplified by a Femto DLPCA-200, then fed into a SRS SR780 signal analyzer. A homemade LabView program is used to measure the spectral current density as a function of signal frequency. Value of the current spectral density at 1 kHz is used for the detectivity calculation.

Supplementary Note 8: Estimation of the intraband diode NETD

Because ultimately this type of detector is designed to make thermal imaging, we have tested the potential of our diode for detection of a cold object (i.e. a hand which surface is around 32.5 °C) in the room environment (T=20 °C).

As can be seen on Supplementary Figure 10a, the blackbody emission of a 32 °C object is mostly negligible in the interband part of the device spectrum and only the intraband part of the spectrum shows a relevant contribution. Supplementary Figure 10b shows the effect of this hand (cold black body) in front of the device, producing a photocurrent of $I_{Ph} = 0.4$ nA. Assuming that the half-angle of view of the detector is defined by the cryostat windows, we have $\alpha = 14°$. Hence the blackbody formula gives:

$$P_{op} = T \times A_d \, \pi \sin^2 \alpha \int_{\lambda_{min}}^{\lambda_{cut}} S(\lambda, T) d\lambda = 77 \, nW$$

Where T is the transmission factor through the cryostat windows (55%), A_d the detector area, and $S(\lambda, T)$ the spectra radiance of the black body at 32.5 °C. The associated responsivity is then $R = I_{ph}/P_{op} = 5.2$ mA/W

Which is a decent fraction of the responsivity measured with the broadband, 650°C blackbody.

The same way, one can compute the NETD of the detector at 80 K with this experiment. Assuming that this photocurrent corresponds to a temperature difference $\Delta T = 12.5 \, K$ (32.5 °C hand *vs* 20 °C background) we have:

$$NETD = \frac{\Delta T}{\Delta I_{photo}/I_n}$$

Where ΔT is the temperature difference, ΔI_{photo} the associated photocurrent signal and I_n the RMS noise of the device. We have $\Delta T = 12.5 \, K$ and $\Delta I_{photo} = 0.4 \, nA$, and the noise spectral density is measured to be 30 $fA/Hz^{1/2}$ under 1 V at 80 K and 1 kHz. Hence with a 1 kHz bandwidth, we have $I_n = 0.9 \, pA$ and $NETD = 35 \, mK$.

Supplementary Note 9: MHz photoresponse of the diode device

The PV device enclosed in a cryostat kept at 80 K, and illumination is provided by a 4.4 µm pulsed QCL. The duration of the QCL pulse is set from 500 ns to 5 µs while keeping the duty cycle to 10%. Bias is provided by a 1.5 V battery equipped with a potentiometer, and current is amplified by a Femto DUPVA 70 GHz amplifier over a 1 kΩ load. Temporal trace is acquired on a Tektronix MDO3102

oscilloscope. Supplementary Figure 11 shows a scheme of the setup as well as temporal traces of MHz QCL pulses resolved by the diode device at 80 K.

Supplementary Note 10: Stability of the diode device

Devices are kept under air-free atmosphere and exposed to air only when being transferred into a cryostat. I-V curves acquired in the dark on a 3-month old sample in the dark shows noticeable change, see Supplementary Figure 12a, mostly a decrease of dark current on the negative bias side.

The photocurrent spectrum of the aged device under zero bias and 80 K (Supplementary Figure 12b) still shows photocurrent contributions from HgTe 6k, HgTe 4k (though fairly reduced compared to older measurements, see Figure 5a) and, more importantly, HgSe in the mid-infrared.

3. Supplementary Table

Supplementary Table 1 Comparison of the performances of IR detector based on epitaxial quantum wells and quantum dots.

Year	Detector type / Active material	Operating wavelength	T_{OP}	Figures of Merit			Comments	Ref.
				R (mA/W)	D* (Jones)	t_{resp}		
2019	HgSe/HgTe Colloidal Nnaocrystals	3.8 µm	80 K	~5	$2{\cdot}10^{9}$	<200 ns	Colloidal QDIP	This work
2018	GaAs/AlGaAs QWIP	9 µm	300 K	300	$1{\cdot}10^{8}$	< ns	Room-temperature detector with patch antenna. Used for heterodyne detection.	1
2013	InAs QD in GaAs matrix	4-7 µm	80 K	30	$1.8{\cdot}10^{9}$	-	p-type QD, 17% quantum efficiency	2
2011	InGaAs/AlGaAs/ GaAs QWIP	5 µm	150 K	-	$2{\cdot}10^{10}$	-	D* > 10^{8} with TEC cooler	3
2010	InAs QD in InGaAs matrix	9 µm	80 K	-	$8{\cdot}10^{10}$	-	Enhanced absorption with 2D plasmonic hole array	4
2007	InAs QD in InGaAs QW on InP	4.1 µm	300 K	50	$6.7{\cdot}10^{7}$	-	Dot-in-well structure<br>35% quantum efficiency	5
2007	InAs QD in InGaAs QW on InP	4.1 µm	120 K	667	$2.8{\cdot}10^{11}$	-	Dot-in-well structure<br>35% quantum efficiency	5
2006	InGaAs QD in InGaP matrix on GaAs	4.7-5.5 µm	77 K	1200	$1.1{\cdot}10^{12}$	-	Low pressure MOCVD grown-QD<br>1% quantum efficiency	6
2006	InP QCD	5.3 µm	150 K	5	$2{\cdot}10^{8}$	-	Quantum Cascade Detector	7

4. Supplementary References

1. Palaferri, D. *et al.* Room-temperature nine-µm-wavelength photodetectors and GHz-frequency heterodyne receivers. *Nature* **556**, 85–88 (2018).
2. Lao, Y.-F. *et al.* InAs/GaAs p-type quantum dot infrared photodetector with higher efficiency. *Appl. Phys. Lett.* **103**, 241115 (2013).
3. Hinds, S. *et al.* Near-Room-Temperature Mid-Infrared Quantum Well Photodetector. *Adv. Mater.* **23**, 5536–5539 (2011).
4. Chang, C.-C. *et al.* A Surface Plasmon Enhanced Infrared Photodetector Based on InAs Quantum Dots. *Nano Lett.* **10**, 1704–1709 (2010).
5. Lim, H., Tsao, S., Zhang, W. & Razeghi, M. High-performance InAs quantum-dot infrared photodetectors grown on InP substrate operating at room temperature. *Appl. Phys. Lett.* **90**, 131112 (2007).
6. Szafraniec, J. *et al.* High-detectivity quantum-dot infrared photodetectors grown by metalorganic chemical-vapor deposition. *Appl. Phys. Lett.* **88**, 121102 (2006).
7. Graf, M., Hoyler, N., Giovannini, M., Faist, J. & Hofstetter, D. InP-based quantum cascade detectors in the mid-infrared. *Appl. Phys. Lett.* **88**, 241118 (2006).
